# SCLC TumorMiner: A genomics platform for small cell lung cancer precision oncology

**DOI:** 10.1016/j.isci.2026.117376

**Published:** 2026-09-03

**Authors:** Fathi Elloumi, Anjali Dhall, Daiki Taniyama, Augustin Luna, Yasuhiro Arakawa, Sudhir Varma, Yanghsin Wang, Anisha Tehim, Mark Raffeld, Kenneth Aldape, Christophe Redon, Roshan Shrestha, William Reinhold, Mirit Aladjem, Jaydira Del Rivero, Nitin Roper, Anish Thomas, Yves Pommier

**Affiliations:** 1 Laboratory of Molecular Pharmacology and Developmental Therapeutics Branch, Center for Cancer Research, NCI, NIH, Bethesda, MD, USA; 2 Computational Biology Branch, National Library of Medicine, NIH, Bethesda, MD, USA; 3 Department of Clinical Pharmacology and Therapeutics, The Jikei University School of Medicine, Tokyo, Japan; 4 HiThru Analytics LLC, Princeton, NJ, USA; 5 Essex Management, LLC, An Emmes Company, Rockville, MD, USA; 6 Cornell University, Ithaca, NY, USA; 7 Laboratory of Pathology, Center for Cancer Research, NCI, NIH, Bethesda, MD, USA

**Keywords:** small cell lung cancer, SCLC, patient clinical and genomics data, SCLC primary and relapse classification, cell surface targets for immune therapies, SCLC TumorMiner web portal

## Abstract

Small cell lung cancer (SCLC) is among the most aggressive malignancies. Unlike many other cancers, it is not represented in The Cancer Genome Atlas, and available datasets are fragmented across institutions, disease stages, and treatment settings. RNA sequencing provides a powerful and cost-effective approach, but the high dimensionality of transcriptomic data and the heterogeneity of patient cohorts pose significant challenges. To address such challenges, we developed SCLC TumorMiner (https://discover.nci.nih.gov/SclcTumorMinerCDB/), which includes 50 tumor samples from relapsed patients at the National Cancer Institute (NCI) and 154 samples from untreated patients at the University of Cologne and Tongji University. SCLC TumorMiner enables molecular classification, genomic pathway analyses, risk stratification, identification of predictive cell-surface biomarkers such as *DLL3* or *TROP2*, and drug-response biomarkers such as *SLFN11*. SCLC TumorMiner illustrates profound differences between untreated and relapsed patient samples. Additionally, “MyPatient”, one of SCLC TumorMiner’s modules, is presented as a medical assistant application prototype.

## Highlights

• SCLC TumorMiner allows transcriptomic, mutation, pathway, and survival analyses
• Marked differences are observed between untreated and relapse tumors
• NMF-based classification provides molecular subtyping of patients with SCLC
• The “MyPatient” module proposes precision therapy guidance

## Graphical abstract

**Figure:**
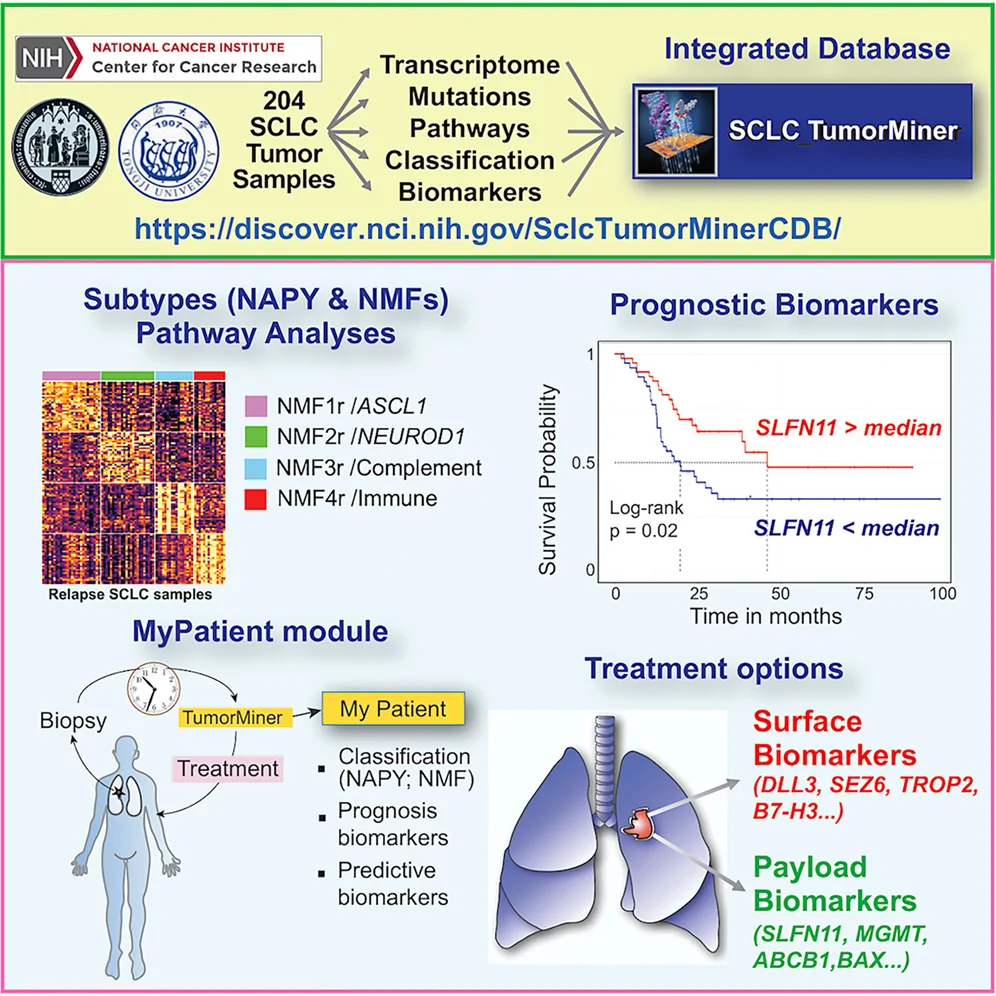

## Teaser

Systems biology; Cancer systems biology; Cancer

## Introduction

Small cell lung cancers are rapidly proliferative tumors often diagnosed with metastases (Extensive-stage; ES-SCLC).^1^ They account for approximately 13%–15% of all lung cancer cases and remain one of the most lethal malignancies. Survival is less than one year for most patients, and 95% of patients die within 5 years despite initial response to cisplatin-etoposide combination therapy. Upon relapse, tumors evolve and become resistant to chemotherapy, reflecting profound changes in their biology and genomic network.

**Figure 1:**
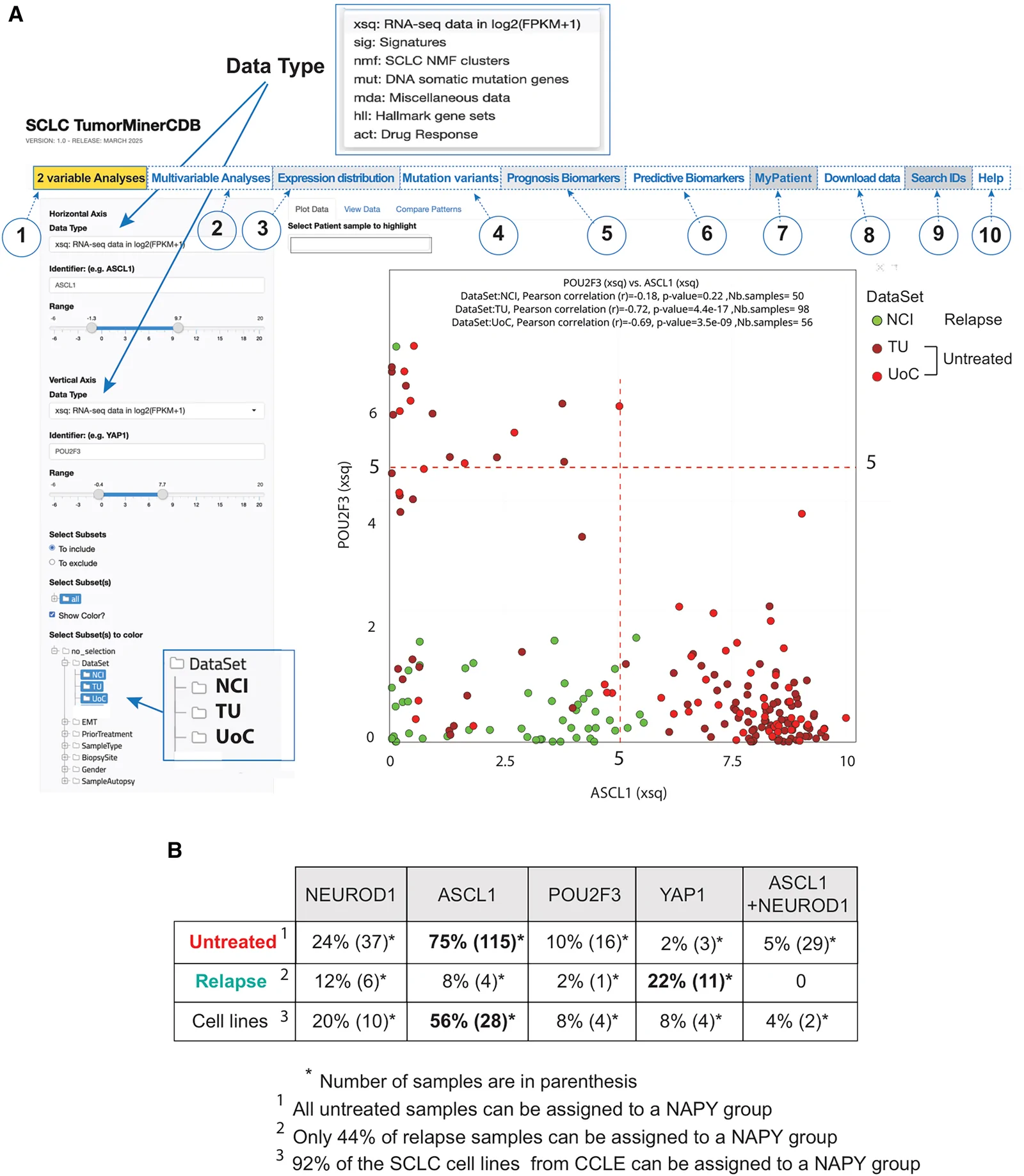
Introduction to SCLC TumorMiner (https://discover.nci.nih.gov/SclcTumorMinerCDB/) (A) Snapshot of the introductory “2 variable Analyses” display (1). Both the horizontal and vertical axes offer a choice of “Data Type” shown in the upper inset. Users must type their choice in the “Identifier” box (here ASCL1 for the horizontal axis and POU2F3 for the vertical axis). Other options include choosing the “Range” for the axes and the “Select Subsets” to “include” or “exclude”. The + sign and folder indicate that further selections can be made, such as choosing the “Dataset” to display. To display more than one dataset, users need to press the Command key while clicking the individual datasets (lower inset). Other features of SCLC TumorMiner include: (2) “Multivariable Analyses” with examples shown in Figure 2; (3) “Expression Distribution” across the 4 Datasets with example shown in Figure S1; (4) “Mutation variants”; (5) “Prognosis Biomarkers” with example shown in Figures 5 and S4; (6) “Predictive Biomarkers” with example shown in Figure 6; (7) “My Patient” to display the genomic characteristics of a selected patient with example shown in Figures 7 and S6. Additional features include the ability to “Download Data” for further analyses (8), as shown in Figures 7, S1, S4 and S5, “Search IDs” (9), as well as a detailed “Help” section (10). (B) NAPY distribution of samples from untreated and relapsed patients and from the SCLC cell lines (https://discover.nci.nih.gov/SclcCellMinerCDB).^4^ Samples were assigned to each NAPY group based on gene expression log2(FPKM+1) > 5.0 (see horizontal and vertical dotted lines in A and justification for the >5.0 threshold in Figure S1).

In addition to mutations of the tumor suppressors *TP53* and *RB1* in 80% of SCLC, selective activation of transcription factors characterizes the disease.^2^^,^^3^ At diagnosis, SCLCs have been divided into two groups. 70–80% are neuroendocrine (NE), driven by two pioneer neurogenic transcription factors, *ASCL1* (Achaete-scute homolog 1) and *NEUROD1* (Neurogenic differentiation factor 1). The remaining non-NE SCLCs are driven by two other transcription factors, *POU2F3* (POU class 2 homeobox 3) and *YAP1* (Yes-associated protein 1). Consequently, SCLCs have been classified into 4 subsets: SCLC-A, SCLC-N, SCLC-P, and SCLC-Y^3^ (NAPY classification^3^^,^^4^). Additionally, an Inflamed/Immune subgroup has been identified as SCLC-I.^5^ These classifications are well supported in untreated tumors and SCLC cell lines^3^^,^^4^ (Figure 1B), but are not routinely applied in clinical practice, where diagnosis and classification rely primarily on neuroendocrine marker immunohistochemistry for synaptophysin (encoded by the *SYP* gene), chromogranin (encoded by *CHGA*), and neural cell adhesion molecule (encoded by *NCAM1*).

Immune checkpoint (PD-L1) inhibitors added to chemotherapy are the standard of care for first-line treatment of ES-SCLC.^6^^,^^7^ However, they only provide a modest overall survival benefit of 2–3 months.^7^^,^^8^ A recent therapeutic breakthrough for SCLC is Tarlatamab®, a bispecific T-cell engager (BiTE®) targeting the Notch ligand cell surface glycoprotein Delta-like ligand 3 (*DLL3*), a transcriptional target of *ASCL1*.^4^ However, responses are heterogeneous, and Tarlatamab® is currently administered without mandatory biomarker-based patient selection.

In parallel, antibody-drug conjugates (ADCs) and CAR-T therapies targeting surface antigens such as *DLL3*, *TROP2* (anti-trophoblast antigen 2 encoded by *TACSTD2*), *SEZ6* (seizure 6-like), carcinoembryonic antigen-related cell adhesion molecules (*CEACAM5* and *CEACAM6*), and *B7-H3* (member of the B7-cell family homolog 3 encoded by *CD276*) are actively being developed. For ADCs in particular, therapeutic efficacy depends not only on target expression but also on payload sensitivity, including replication stress-associated vulnerabilities such as expression of Schlafen 11 (*SLFN11*) and Ki67 (encoded by *MKI67*).

With the advancement of high-throughput genomic sequencing methods, bulk RNA sequencing (RNA-seq) has emerged as a well-established, sensitive, and clinically usable method for transcriptome analyses. However, even when only applied to protein-coding genes, it generates around 20,000 gene expression data points per sample, which are difficult to analyze instantaneously in a clinical setting. To address this challenge, we developed SCLC TumorMiner, integrating 204 SCLC patient samples. Fifty samples were collected from patients at relapse in our NCI clinic, and 154 are from the published datasets of untreated patients from the University of Cologne (UoC)^2^ and Tongji University (TU)^9^ (Tables 1 and S1). The architecture and interface of TumorMiner are based on our SCLC CellMinerCDB website comprising the genomics and drug responses of 118 SCLC cell lines (https://discover.nci.nih.gov/SclcCellMinerCDB).^4^ Thus, SCLC TumorMiner and SCLC-CellMinerCDB can be readily used to compare tumor samples with cell lines.

**Table 1:** SCLC samples and datasets

Number of Samples		Untreated		Relapse	Total
		UoC	TU	NCI	
		56	98	50	204
Genomics	RNA-seq	56	98	50	204
	WES	0	98	16	114
Prior treatment	yes	0	0	50	50
	no	52	98	0	150
	unknown	4	0	0	4
Biopsy site	lung	55	98	7	160
	liver	0	0	21	21
	lymph node	0	0	10	10
	pleura	1	0	1	2
	adrenal gland	0	0	4	4
	mediastinal mass	0	0	3	3
	others	0	0	4	4
Tumor stage	stage 1	25	30	0	55
	stage 2	7	26	0	33
	stage 3	17	41	2	60
	stage 4	7	1	48	56

Here we provide a tutorial and demonstrate how SCLC TumorMiner allows instantaneous: 1/Transcriptome and mutation analyses of patient samples; 2/Molecular pathway analyses; 3/Patient sample classification based on NAPY and/or NMF (non-negative matrix factorization); and 4/Identification of prognosis and predictive biomarkers for targeted therapies.

## Results

### Introduction to SCLC TumorMiner and brief tutorial

SCLC TumorMiner uses the same architecture as our six-cancer cell line CellMiner websites (https://discover.nci.nih.gov/). Thus, users familiar with SCLC-CellMinerCDB (https://discover.nci.nih.gov/SclcCellMinerCDB/) will have no difficulty navigating back and forth between the tumor samples and cell lines databases.^4^

Figure 1 (https://discover.nci.nih.gov/SclcTumorMinerCDB/) introduces the website as a snapshot of the default page display. The upper part shows the tabs for each of the tools and applications. They are numbered 1–10 for convenience in the figure. The “2 variable Analyses” tab (numbered “1” in Figure 1A) allows the display of different data types or parameters on each of the axes: “xsq” (RNA-seq), “sig” (Signatures), “mut” (gene mutations), “mda” (Miscellaneous data), “hll” (Hallmark gene sets) and “act” (Drug Response). For instance, plotting *ASCL1* expression vs. neuroendocrine (NE) signature (Figure S1A) shows their expected high correlation within each of the 3 datasets (NCI, UoC, TU).

Two-variable analysis data can be downloaded as Tab-delimited files for further exploration. Figure 1B compares the expression of *NEUROD1*, *ASCL1*, *POU2F3,* and *YAP1* across datasets. It demonstrates the reduced *ASCL1* and *POU2F3* expression associated with increased *YAP1* expression in the relapse samples (Figure 1A). As a result, the assignment of samples to each of the NAPY groups can only be applied to 44% of the relapse samples while it discriminates all the samples from untreated patients^3^ and 92% of the cell lines from SCLC-CellMinerCDB^4^ (Figure 1B).

**Figure 2:**
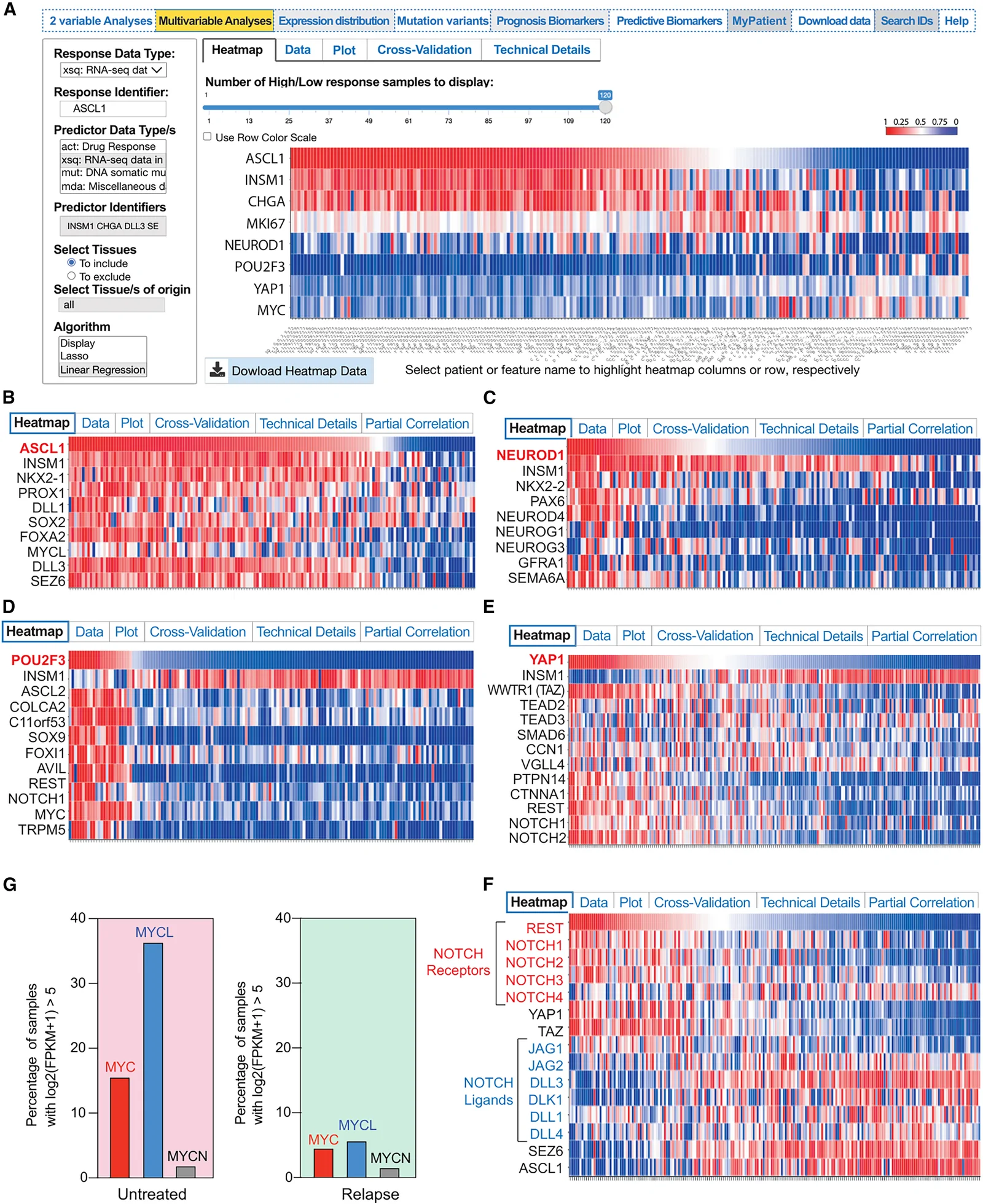
Multivariable Analyses showing co-regulation of the neuroendocrine (*INSM1, CHGA*), NAPY (*NEUROD1, ASCL1, POU2F3* and *YAP1*), NOTCH (*NOTCH1, NOTCH2, REST*) and MYC (*MYC, MYCL and MYCN*) pathways (A) Default display for the “Multivariable Analyses” tab. Color scale goes from red to blue to reflect expression levels for each gene (rows) and patients (columns). Users can choose different data types (upper inset of A) and then type the “Response Identifier” to display as the first row on the heatmap. “Predictor Identifier (s)” need to be typed with a space between each. “Tissues of origin” can be selected. The default algorithm (bottom left) is “Display”. Users can also perform Linear and Lasso analyses such as in CellMinerCDB (https://discover.nci.nih.gov/cellminercdb/). (B–E) Multivariable analyses for the NAPY genes used as “Response Identifiers”. *INSM1* is included in each analysis as an NE biomarker. B–D only include the samples from the databases of untreated patients. (F) Multivariable analysis for the Notch pathway. (G) Differential expression of the 3 MYC genes in samples from untreated and relapsed patients. Linear regression analyses for each of the “Response Identifiers” (in red, 1^st^ rows in B–F) show that the “Predictor Identifiers” are highly significant (Figure S2C).

The “Multivariable Analyses” tab (labeled “2” in Figure 1A) shows as default display the heatmap of *ASCL1* expression (xsq: RNA-seq) vs. the expression of key genes driving SCLC: *INSM1* and *CHGA* encoding immunohistochemistry NE markers, *MKI67* (encoding Ki67 used as proliferation marker), and the SCLC pioneer transcription factors *NEUROD1*, *POU2F3* and *YAP1*, and the oncogene *MYC* (Figure 2A). Each row displays the selected genes, and the columns represent each of the 204 individual patient samples. Users can explore different inputs and outputs by entering gene names in the “Response Identifier” or “Predictor Identifiers” windows (left panels of Figure 2A). Examples of “Multivariate Analyses” for the NAPY genes are shown in Figures 2A–2E and will be discussed in the next section.

Under the “Multivariate Analyses” tab, in addition to the default visualization set up by default as “Display”, users can choose the “Linear Regression” tab (found at the bottom left corner of the website under “Algorithm”; Figure 2A). This feature computes the correlation between each of the “Predictive Identifiers” and the “Response Identifier”. Figures S2A and S2B show the results for *ASCL1* for the genes entered in Figure 2B. The “Plot” and “Technical Details” options illustrate the highly significant value of the combined expression of *NKX2.1*, *FOXA2*, *DLL3,* and *SEZ6* as predictors of *ASCL1* expression in the combined datasets (UoC and TU) of the untreated patients (Figures S2A and S2B). Users can also choose “Lasso” (Least Absolute Shrinkage and Selection Operator), a machine-learning and statistical method to identify genomic biomarkers predicting the expression of “Response Identifiers”. For *YAP1* expression, the top gene identified in the relapse samples is the Neuron Restrictive Silencing Factor *REST*, a key effector of the Notch pathway (Figure S2D).^4^

The “Expression Distribution” tab (labeled “3” in Figure 1A) displays the expression of any chosen gene across the 3 datasets (the two untreated TU and UoC datasets and the relapsed patients from the NCI dataset). *SLFN11* expression is displayed by default on the website (Figure S3A). Its reduced expression in the relapse samples is consistent with the resistance of relapsing patients to DNA-targeted chemotherapies. Users can also display and download data for any gene from each of the 3 datasets by typing the gene HUGO name in the “Gene symbol” box. Such analyses for the housekeeping genes *GADPH*, *ACTB* (β-actin), or *B2M* (β2-macroglobulin) show high expression and lack of significant expression differences between untreated and relapse samples, suggesting limited batch effect for highly expressed genes (>5 in at least one dataset).

The “Mutation Variants” tab (labeled “4” in Figure 1A) displays up to 5 genes at a time. Colors reflect the different types of mutations, including: Missense_Mutation, Nonsense_Mutation, Frame_shift_Ins, Frame_Shit_Del, In_Frame_Del, Multi_Hit. Variants can be viewed and downloaded with the “View variants” tab (Figure S3B). As expected, *TP53* is the most frequently mutated gene (81% of the 114 samples with whole-exome sequencing data). The second most frequently mutated gene is *TTN,* encoding Titin. This is not surprising, as Titin is the largest human protein and SCLC has a high mutation burden. *RB1* shows an overall mutation frequency of 55% (Figure S3B). By computing the copy number (Ploidy) values for *RB1* for the TU and NCI SCLC (114) samples and using an *ad hoc* rule (samples with <0.05 ploidy for full depletion and between 0.05 and 1.85 for partial depletion), percentages for full and partial depletions are 62.8% and 17.7%, respectively (Table S2). Inactivation of *SLFN11* was observed in only 4% of samples (Figure S3B), consistent with epigenetic inactivation as the main mechanism for suppressing *SLFN11* expression and conferring chemoresistance.^10^

The “Prognosis Biomarkers”, “Predictive Biomarkers” and “MyPatient” tabs (numbered 5, 6, and 7 in Figure 1A) will be discussed in the remaining sections of the manuscript, with examples displayed in Figures 5 and 6.

### Genomic networks and pathway explorations

We previously reported^4^ in SCLC cell lines, the expression of genes of a given pathway is cross-correlated (see Figure 5 in^4^). The main pathways for SCLC comprise the neuroendocrine (NE) pathways driven prominently by *ASCL1* and less frequently by *NEUROD1*, and the non-NE pathways driven by *POU2F3* and *YAP1*, the *MYC* genes, and the Notch pathway.^3^^,^^4^ As in the SCLC cell lines (https://discover.nci.nih.gov/SclcCellMinerCDB/),^4^ the group of NE-SCLC prominently expresses *INSM1* and *CHGA,* encoding routinely used immunohistochemistry biomarkers (Figure 2A). As expected, *ASCL1* expression is highly correlated with *INSM1* and *CHGA*. Figure 2A also shows that multiple samples express both *ASCL1* and *NEUROD1*, and a significant overlap between *YAP1*, *POU2F3,* and *MYC* expression.

Analysis of the *ASCL1* coexpression network in the samples from untreated patients (Figure 2B) confirms^4^ that most samples expressing *ASCL1* coexpress *INSM1* (highly significant correlations in the “2 variable analysis”). As expected, the transcriptional coactivators of *ASCL1*, *NKX2-1* and *PROX1*^4^^,^^11^ show coexpression (confirmed in the “2 variable analysis”). *DLL3*, the downstream transcriptional target of ASCL1, is highly expressed in approximately two-thirds of the samples, with high correlation with *ASCL1* expression (confirmed in the “2 variable Analyses”). *SEZ6*, another *ASCL1* target gene and an emerging cell surface target for antibody-drug conjugates, is also highly correlated with both *ASCL1* and *DLL3* expression (Figure 2B). Of note, *SEZ6* expression is highly correlated with *SEZ6L* (SEZ6 homolog-like) despite different chromosome locations (17q11.2 and 22q12.1, respectively), suggesting that both genes function in the *ASCL1* pathways.

Transcriptome analysis of the *NEUROD1* genomic network (Figure 2C) shows that *NEUROD1* expression only accounts for a fraction of the NE samples. Indeed, many samples show high *INSM1* expression without high *NEUROD1* (Figure 2C, compare the 1^st^ and 2^nd^ rows). *NKX2-2* [*NK2* transcription factor related, locus 2 (Drosophila)], which is involved in the morphogenesis of the central nervous system (CNS) and a known regulator of *NEUROD1* in pancreatic islet cells together with the Neurogenin genes (*NEUROG1* and *NEUROG3*),^12^ is co-expressed with *NEUROD1* (Figure 2C). The coexpression of *PAX6* (Paired Box Homeotic Gene-6) encoding a transcription factor for the development of the eyes, nose, pancreas and CNS, is logical as *PAX6* controls *NEUROD1* expression.^13^ Using the “2 variable Analysis” and the “Compare Patterns” tools of SCLC TumorMiner (Figure 1A), we discovered coexpression of two genes encoding cell surface proteins and potential targets for immune therapies, *GFRA1* (Glial Cell Line-Derived Neurotrophic Factor Receptor Alpha) and *SEMA6A* (encoding one of the semaphorin)^14^ (Figure 2C). Like the samples from untreated patients, the SCLC cell lines also express significant levels of *SEMA6A* with high correlation with *NEUROD1* (*p* = 0.57 with *p*-value = 2.7e−11).^4^ Whether *NEUROD1* directly drives *GFRA1* and *SEMA6A* expression remains to be determined.

The *POU2F3* network shows the expected inverse expression between *POU2F3* and *INSM1* (Figure 2D, compare the 1^st^ and 2^nd^ rows), as the *POU2F3* cells are derived from the Tuft cells of the bronchial epithelium and are non-NE.^15^^,^^16^^,^^17^ Figure 2D shows that the tumor samples expressing *POU2F3* coexpress 4 transcription factors known to drive *POU2F3* expression^15^^,^^18^: *ASCL2, COLCA2* (also known as *OCAT-2* or *POU2AF3*), *C11orf53* (also known as *OCAT-1* or *POU2AF2*), and *SOX9*. *POU2F3* is also highly correlated with the Tuft-ionocyte progenitor-specific genes *FOXI1* and *AVIL*,^16^^,^^17^ as well as with *TRPM5* (Transient Receptor Potential Cation Channel Subfamily M Member 5), which encodes a cell surface ion channel protein highly expressed in bronchial Tuft cells and involved in immune defense.^19^ This suggests that *TRPM5* could be considered a cell surface target for SCLC-P tumors. Figure 2D also shows that the *POU2F3* network/pathway is coordinated with the Notch pathways, as *POU2F3* expression is highly correlated with *REST* and its transcriptional activator *NOTCH1* (validated in the “2 variable analysis” for the untreated samples). *MYC* expression is also highly correlated with *POU2F3*, consistent with independent reports.^9^^,^^17^^,^^20^ These findings suggest that the expression of the 11 genes displayed in Figure 2D could be used as predictors for *POU2F3* expression in tumor samples (Figure S2C) and for classifying a given tumor sample as SCLC-P.

Analysis of the *YAP1* network across our 3 datasets shows that samples with high *YAP1* expression tend to lack *INSM1* expression (Figure 2E). By contrast, *TAZ* (encoded by *WWTR1*), the transcriptional co-activator partner of YAP1, is co-expressed with *YAP1*, consistent with the SCLC cell lines (Figure 5 in^4^). Similarly, *TEAD2* and *TEAD3*, the downstream transcription factors of *YAP/TAZ,* are co-expressed with *YAP1*, as well as *YAP1*’s transcriptional targets *SMAD6*, *VGLL4* and *PTPN141*.^4^ Figure 2 also shows that the cell adhesion genes *CCN1*, *CTNNA1* and *CTNNA2* are coexpressed with *YAP1*. Consistent with the SCLC cell line data (see Figures 5C and 5D in^4^), Notch pathway activation (*REST*, *NOTCH1* and *NOTCH2*) is associated with the SCLC-Y samples. Together, the expression of the 12 genes displayed in Figure 2E significantly predicts *YAP1* expression both in the SCLC tumors and cell lines (Figure S2C) and therefore could be used to classify a given tumor sample as SCLC-Y (Figure S2D).

Using *REST* expression as a reporter for the Notch pathway (Figure 2F), we found coexpression of *REST* with the 4 Notch receptors, *NOTCH1-4,* and with *YAP/TAZ*, consistent with activation of the Notch pathway by *YAP1*-mediated transcription.^21^ By contrast, expression of the Notch ligands (*JAG1/2*, *DLL3*, *DLL1*, *DLL4* and *DLK1*) is significantly suppressed in the Notch receptor-positive samples and co-expressed with *DLL3* and *ASCL1* (Figure 2F). These findings support the conclusion that the *YAP/NOTCH/REST* network controls the NE fate of SCLC cells^21^ and vice versa, as *DLL3* suppresses Notch signaling in SCLC.^22^

Figure 2G shows *MYC* pathway activation in 52% of the untreated patient samples, where *MYCL* dominates (36.4% of the samples). *MYCL* expression also appears linked with *ASCL1* expression (SCLC-A) (Figure 2B). By contrast, *MYC* is only highly expressed in a relatively small fraction of the untreated patient samples (15.6%) and primarily in the SCLC-P samples (Figure 2D).^23^ Consistent with the fact that *POU2F3* disappears in the relapse samples and that *ASCL1* expression is also attenuated in those samples, the expression of both *MYCL* and *MYC* appears markedly reduced in the samples of patients at relapse (Figure 2G).

The conservation of each of the 5 networks described above between tumor samples and cell lines (Figure S2C) implies their predictive value as genomic signatures for tumors driven by *ASCL1, NEUROD1, POU2F3, YAP1* and NOTCH.

### NAPY classification of SCLC tumors

To identify highly expressed genes, we first analyzed the gene expression distribution for all genes across the datasets (Figure S1B). The 90-percentile expression value cutoff selected log2(FPKM+1) values >4 for both the untreated and relapse samples. To further increase stringency, we set up a threshold value log2(FPKM+1) > 5 for high gene expression (red lines in Figures 1A and S1A–S1C, and shaded areas in Figures S1B and S1C). We also compared the expression of canonical housekeeping genes in the untreated and relapse datasets and found their expression well above the threshold of 5 and relatively comparable between the untreated and relapse datasets, suggesting limited batch effect (Figure S1C).

Using RNA-seq expression levels >5 [log2(FPKM+1)], we could unambiguously assign all the samples from the untreated patients to the NAPY classification (Figure 1B). However, less than half (44%) of the samples from relapse patients with NCI could be classified, with 22% as high *YAP1* (Figure 1B). This is notably higher than for the untreated patients (22% vs. 2% *YAP1* high; Figure 1B). Notably, based on the same cut-off [log2(FPKM+1)>5], the SCLC cell lines could be classified like the untreated samples, which is consistent with the fact that most SCLC cell lines have been derived from patients before treatment.^24^^,^^25^

**Figure 3:**
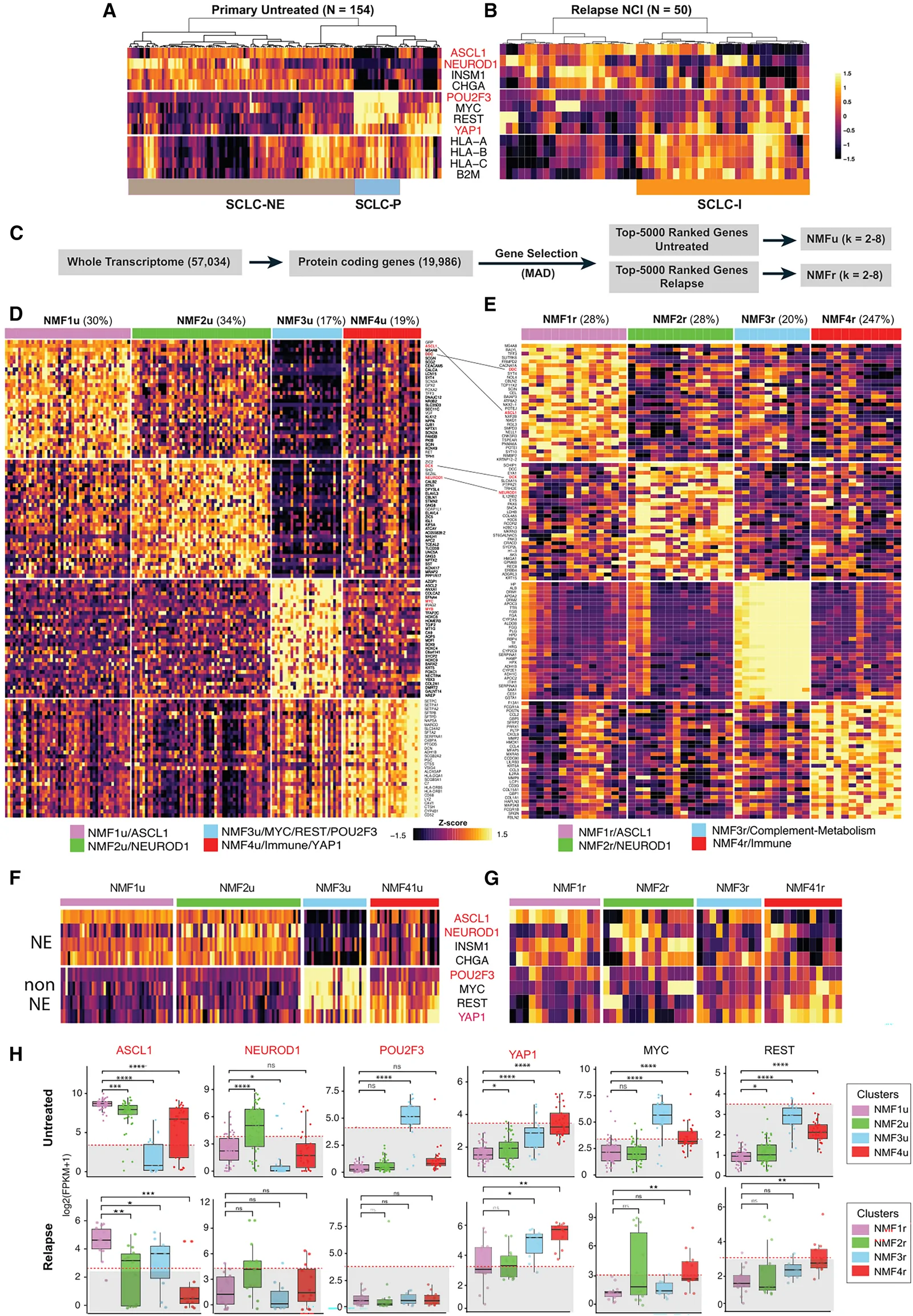
Classification of the untreated (primary) and relapse 204 SCLC samples comparing untreated (UoC and TU datasets) and relapse samples (NCI) (A and B) Hierarchical clustering of the untreated and relapse samples based on the expression of the four genes of the NAPY classification (*NEUROD1*, *ASCL1*, *POU2F3* and *YAP1* in red) and of genes reflecting commonly used biomarkers for clinical neuroendocrine (NE) classification (*INSM1* and *CHGA*) as well as *MYC*, *REST* and 4 exemplary innate immune genes (*HLA-A*, *HLA-B*, *HLA-C,* and *B2M*). (C) Flow chart summarizing the NMF approach for selecting 5,000 protein-coding transcripts based on MAD (Median Absolute Deviation) for the untreated and relapse samples. (D and E) Display of the NMF clusters for the untreated and relapse samples (D and E, respectively). The percentages of samples within each NMF are indicated in parentheses. NMFs for untreated and relapse are abbreviated NMFu and NMFr, respectively. The rows show the top 30 genes for each NMF with log fold-change >1 and *p* value <0.05. Genes that are common across untreated and relapse samples for the NMF clusters are indicated by the oblique lines. (F and G) NMF distribution of the canonical NAPY genes (red) and reference NE genes, as well as *MYC* and *REST.* (H) Boxplots compare the differential expression of the NAPY genes, *MYC,* and *REST* across the 4 untreated (top) and relapse NMFs (bottom). Statistical comparisons were performed by Student’s *t* test. ∗∗∗∗ indicates *p* < 0.001; ∗∗*p* < 0.01; and ∗*p* < 0.05. Shaded areas represent low expression (log2(FPKM+1) <3).

To classify the samples from relapsed patients, we first attempted to extend the gene list for cluster analyses by adding to the 4 NAPY genes (highlighted in red in Figures 3A and 3B), the two NE genes (*INSM1* and *CHGA*), *MYC*, *REST,* and 4 innate immune genes of the major histocompatibility complex (MHC): *HLA-A*, *HLA-B*, *HLA-C,* and *B2M* as reporters of an inflamed signature (SCLC-I).^5^ This 12-gene algorithm classified the samples of untreated patients into 2 groups (Figure 3A; see annotations under the heatmap): SCLC-NE (≈70% of the samples) and SCLC-P with high *POU2F3*, *MYC*, *REST*, and, to a lesser extent, *YAP1 e*xpression. The inflamed group with high MHC expression (*HLA-A/B/C* and *B2M*) was found to be diffuse (≈50% of the patient samples), with a tendency to be excluded from the SCLC-NE group. For the samples of relapsed patients, the 12-gene signature only clustered the samples based on the MHC genes (≈50% of the samples; SCLC-I) (Figure 3B). To overcome the difficulty of classifying the samples from patients at relapse using the NAPY classification, we performed non-negative matrix factorization (NMF) analyses.^5^

### NMF classification of SCLC tumors

To perform these NMF analyses, we used the top 5000 most variably expressed protein-coding genes separately in the untreated (UoC and TU) and relapse (NCI) datasets (Figure 3C and Tables S3 and S4). Among those genes, 64.4% are common to the untreated and relapse samples (Figure S4A). Cophenetic and Silhouette scores showed that 4 clusters gave a satisfactory division of the samples (Figures S4B and S4C).

To develop open-access NMF signatures, we first selected genes with a 2-fold selectivity compared to the genes in the other 3 NMF groups. This led to a list of genes in each NMF group. For the relapse samples, we selected 181 genes for NMF1r, 58 for NMF2r, 342 for NMF3r, and 215 for NMF4r (Table S5). For the untreated samples, we obtained 54 genes for NMF1u, 137 for NMF2u, 85 for NMF3u, and 386 for NMF4u (Table S6). At the end, we obtained 662 and 795 genes for the untreated and relapse cohorts, respectively (see STAR Methods section for details). The top-30 genes for each of the NMFu and NMFr clusters are displayed in Figures 3D and 3E, respectively. Notably, only a few genes are common in the NMF clusters of untreated and relapse patient samples (see diagonal lines between panels D and E in Figure 3 and see Tables S5 and S6).

**Figure 4:**
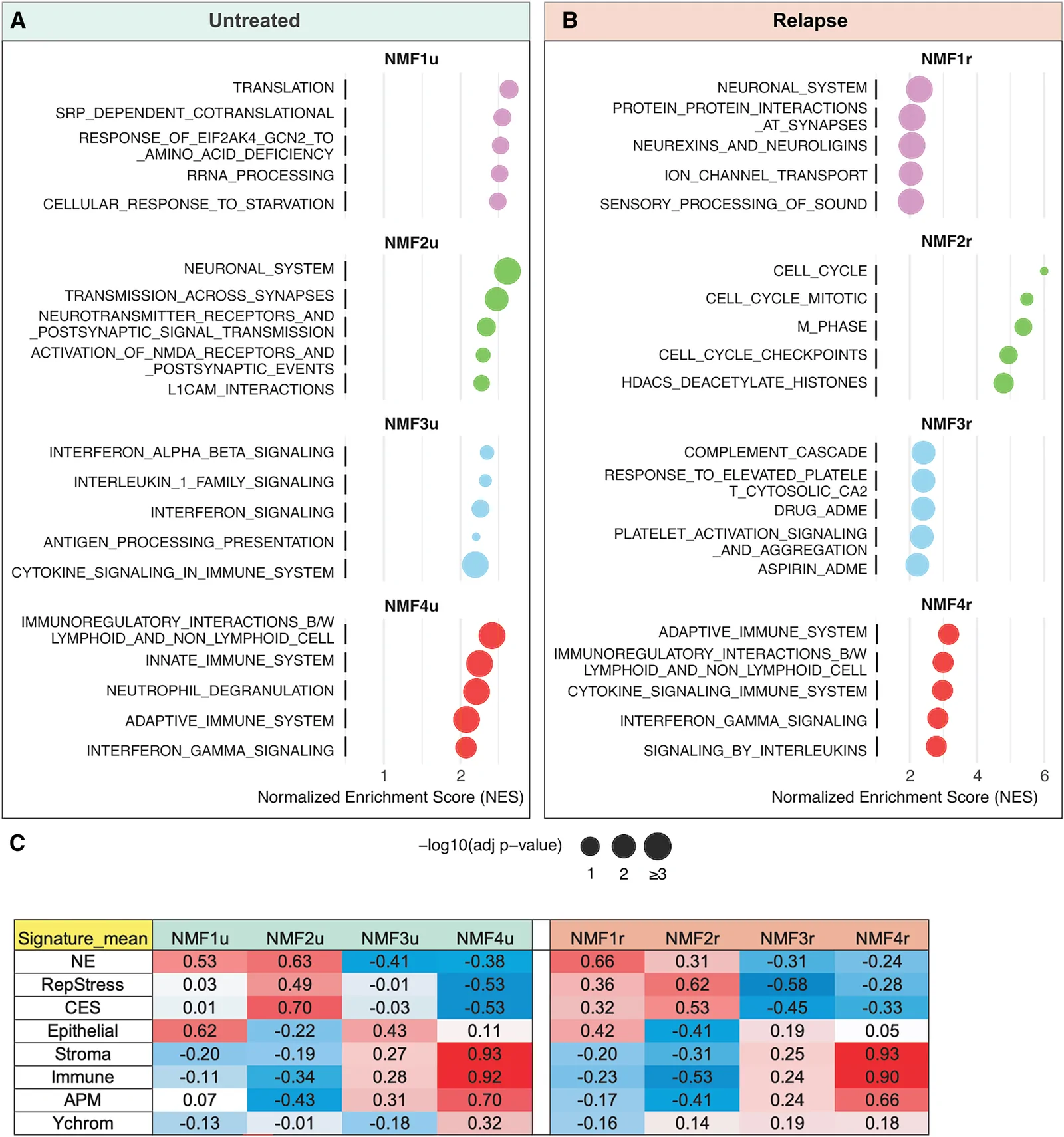
Genomic signatures associated with each NMF from the untreated and relapsed patient biopsy samples (A and B) Reactome pathway analyses for untreated and relapse samples. (C) Correlation between each of the NMF’s and the eight genomic signatures of SCLC TumorMiner for the samples from untreated (left) and relapse (right) patients. Correlations were obtained using the “2 variable Analyses” of the SCLC TumorMiner website (“Plot Data” or “View Data” tabs for each of the NMFs on the horizontal axis). For the untreated samples, the corresponding NMF’s were computed after limiting the analysis using “Select Subsets” for the untreated Datasets: TU + UoC. Conversely, for the relapse samples, analyses were done using the NCI samples.

Pathway analysis shows that NMF1u consists of genes involved in translation and RNA metabolism (Figure 4A) in addition to *ASCL1* (Figures 3D, 3F, and 3H). The NE enrichment of NMF1u is easily visible through the mapping of *ASCL1*, *NEUROD1*, *INSM1,* and *CHGA* in the NMFu clusters (Figure 3F). NMF2u also shows strong NE enrichment (Figures 3F and 4A) and includes *NEUROD1* (Figure 3D). NMF3u includes *POU2F3* (Figures 3F and 3H) and immune and interleukin pathways (Figure 4A) as well as the MYC pathway (Figure 3D; *MYC* and *MYB* annotated in red). NMF4u partially extends to NMF3u and shows positive enrichment for immune, cytokine, and interferon pathways (Figures 3D and 4A). It is enriched for *YAP1* (Figures 3F and 3H).

Pathway analyses for the 4 relapse NMF clusters show that NMF1r includes genes and pathways related to NE differentiation (Figures 3E and 4B) as well as *ASCL1* (Figures 3G and 3H). NMF2r comprises *NEUROD1* (Figures 3E and 3H) and is enriched for cell cycle genes (Figure 4B). NMF3r includes genes involved in metabolism and complement (Figure 4B). It also includes *YAP1* (Figures 3G and 3H). NMF4r consists specifically of immune genes (Figures 3E and 4B) and some *YAP1*-expressing samples (Figures 3G and 3H). The complete pathway enrichment results for untreated and relapse SCLC samples are provided in Tables S7 and S8, respectively.

### The NMF clusters reflect the SCLC TumorMiner molecular signatures

Next, we explored the correlations between the NMF and eight transcriptional signatures provided in SCLC TumorMiner: neuroendocrine (NE), replication stress (RepStress), Chromosome Centromere and Kinetochore score (CES), epithelial, stroma, immune, antigen presenting machinery (APM), and Ychrom (Y chromosome), which were computed as the mean expression of the genes included in each signature (see STAR Methods section).

Figure 4C tabulates the Pearson’s coefficient correlations between each NMF and the 8 signatures, with the colors reflecting the direction and intensity of the correlations (red for positive and blue for negative). Consistent with the top gene signatures and the Reactome pathway analyses (Figures 4A and 4B), the NMF1u, NMF2u, NMF1r and NMF2r signatures are highly correlated with the NE signature as well as with the RepStress and CES signatures. They are also significantly devoid of the Immune, APM, and Stroma signatures (Figure 4C). Conversely, NMF3u, NMF3r, NMF4u and NMF4r are enriched for the Immune, APM, Stroma and epithelial signatures and lack the NE, RepStress and CES signatures. Together, these results demonstrate the functional significance of the NMF classifications for SCLC.

### External validation of the NMFs

To evaluate and validate our NMF-based gene signatures using independent datasets, while avoiding batch effect correction with the training data, we computed NMF scores for each sample based on the signature genes. We then clustered samples using the top 30 most differentially enriched genes and assessed whether the resulting patterns were consistent with those observed in the training datasets. We first performed validation using RNA-seq data from two external untreated SCLC cohorts, GSE60052^26^ (*n* = 79) and IMpower133^8^ (*n* = 271). As both cohorts consist of treatment-naïve samples, we applied the NMFu untreated signatures for clustering and validation (Table S6). As shown in Figures S5A and S5B, the top 30 most differentially enriched genes were consistent with those identified in our untreated training SCLC cohort. Neuroendocrine (NE) markers, including *ASCL1, NEUROD1, INSM1* and *CHGA*, as well as non-NE markers such as *POU2F3, YAP1, MYC* and *REST*, exhibited distinct and consistent enrichment patterns across clusters in both external cohorts. These findings support the biological relevance and reproducibility by identifying the same 4 NMF signatures for untreated samples in these two other external datasets.

Next, we sought to validate the NMF relapse signature using the Wagner dataset,^27^ which includes 22 samples from 18 patients with relapsed SCLC patients. As this dataset contains two technical batches (RNA-seq protocols: polyA and total RNA; both derived from frozen samples), we applied batch correction as described in the original study. As shown in Figure S6A, the top 30 most differentially enriched genes were consistent with those observed in three of our relapse SCLC clusters: NMF1r, NMF2r, and NMF4r. We did not identify a cluster corresponding to NMF3r of the NCI training dataset, which is associated with complement and metabolic pathways. To further investigate the consistency of the 4 NMF relapse clusters, we applied the NMF method on the Wagner dataset using the same set of input genes (∼5000 genes) (as with the NCI dataset) and computed the correlation between the metagenes from the NCI basis matrix and the equivalent matrix generated for the Wagner dataset. Figure S6C shows the correlation of Wagner metagene groups with the NCI metagene groups. Wagner W3 group only had low correlation to the NCI groups (*r* = 0.29), whereas other subgroups had correlations greater than 0.6 (for W1, W2 and W4). This discrepancy may be due to differences in sample composition, including the absence of liver metastasis samples and variation in biopsy sites between the NCI and Wagner datasets (Figure S6B).

### Prognosis biomarkers and risk factors

**Figure 5:**
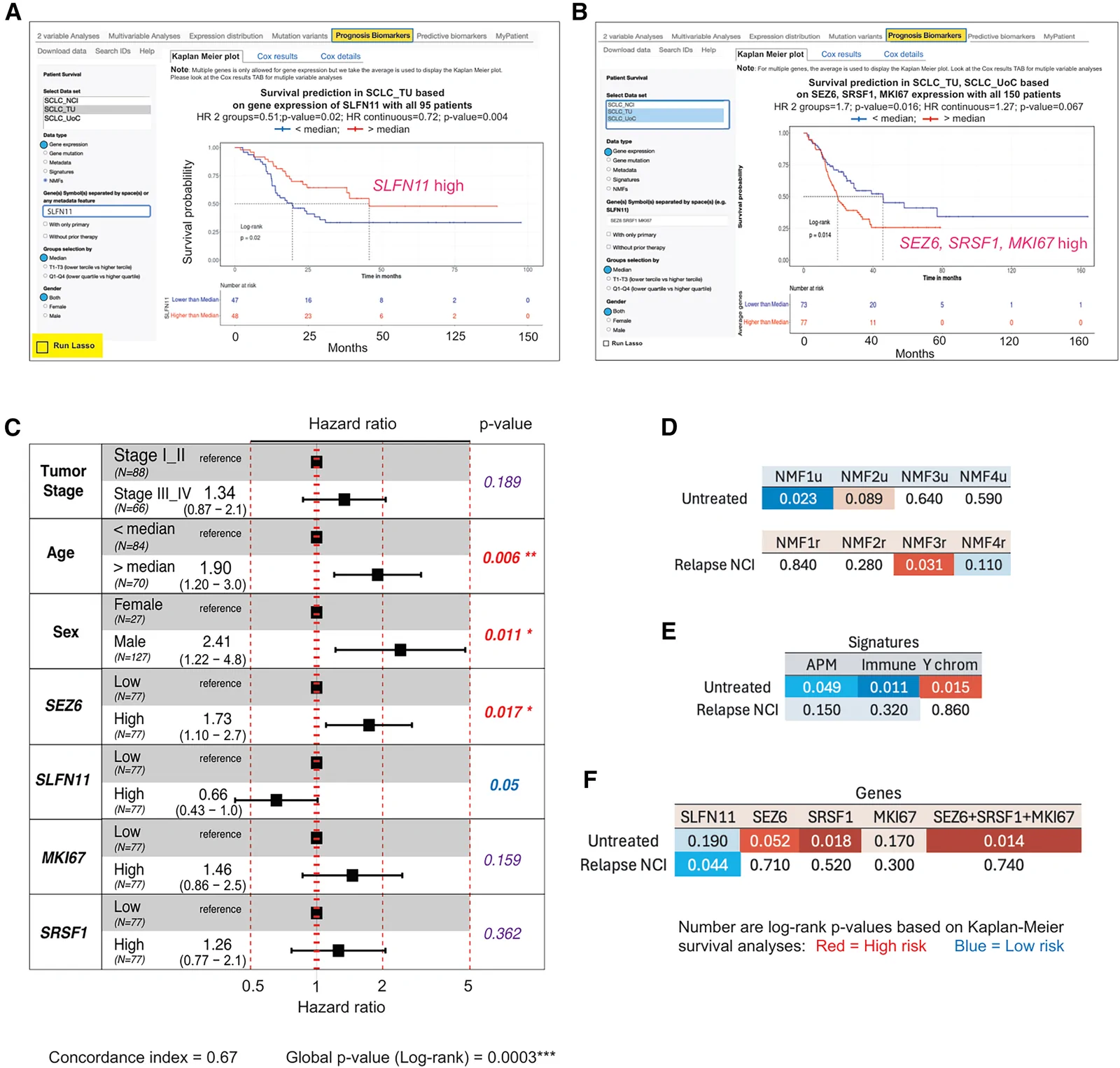
Exploration of prognosis biomarkers (A) and (F) are screen snapshots of the SCLC TumorMiner website. (A) High *SLFN11* expression is a good prognosis biomarker for the untreated TU Dataset. (B) Combined expression of *SEZ6*, *SRSF1,* and *MKI67* is a significant risk factor for the combined untreated datasets (TU and UoC). (C) Multivariate survival analysis including *SEZ6, SLFN11*, *SRSF1*, *MKI67* expression, age, tumor stage and sex for the untreated patients (TU and UoC datasets). (D) Tabulated results for the prognostic value of each NMF cluster. (E) Prognostic value of 3 of the 8 signatures of SCLC TumorMiner. (F) Prognostic value of *SLFN11*, *SEZ6, MKI67,* and combined expression of *SEZ6*, *SRSF1,* and *MKI67* as prognostic biomarkers for the untreated and relapse datasets. Numbers (D–F) are log rank *p*-values based on Kaplan-Meier survival analysis, and colors highlight significant values (*p* < 0.05), with low-risk parameters in blue and high-risk in red.

**Figure 6:**
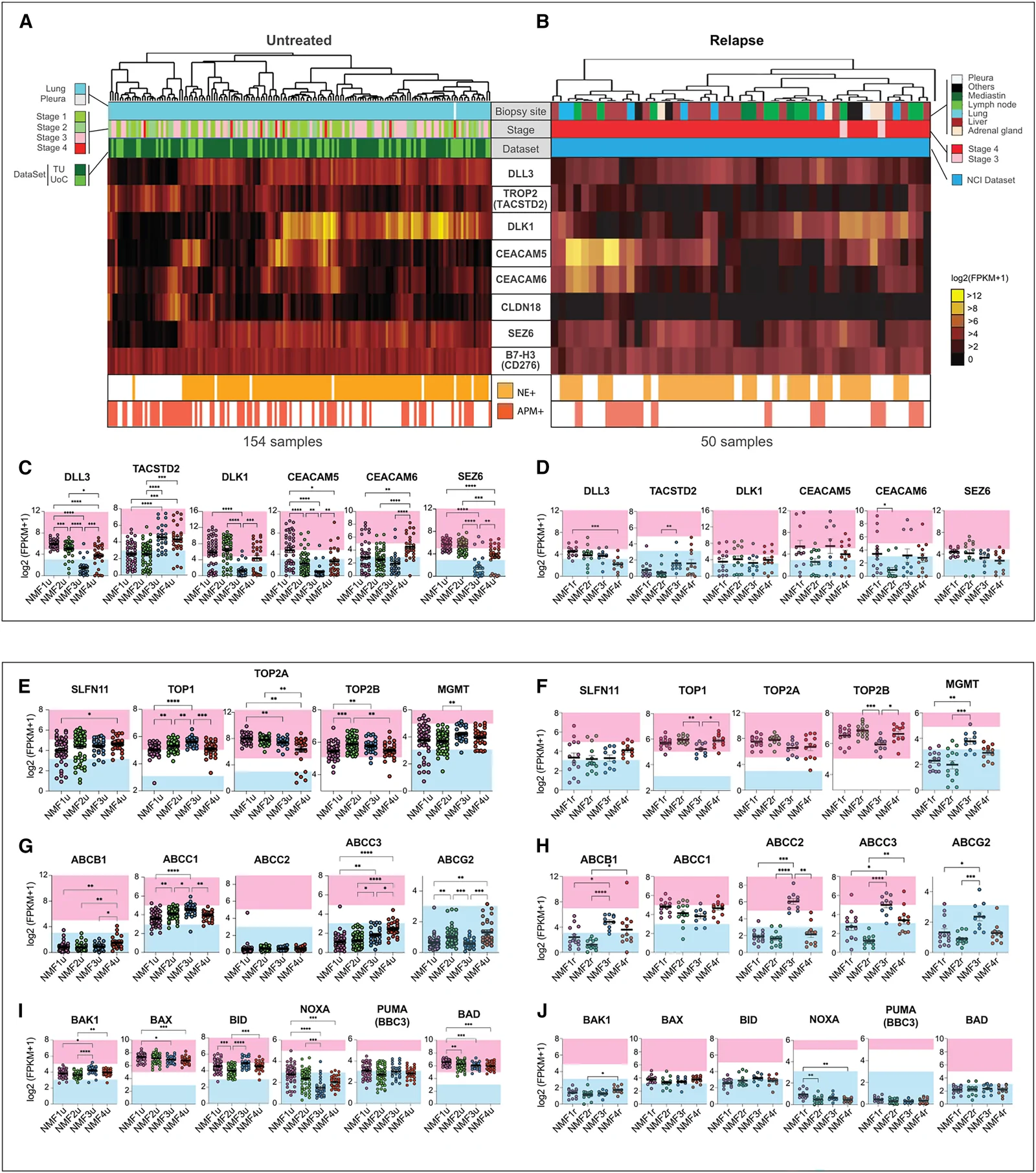
Predictive biomarkers with focus on ADCs The left panels (A, C, E, G, and I) are specific to untreated patients whereas the right (B, D, F, H, and J) are specific to patients after relpase. (A and B) Heatmaps show the differential expression of 8 selected ADC targets. Samples were clustered based on the ADC target surface receptor genes listed between A and B. *DLL3* and *TROP2* (*TACSTD2*) are both FDA-approved targets. Expression of *DLK1*, *CEACAM5*, *CEACAM6*, *CLN18*, *SEZ6*, and B7H3 is displayed because of its selective expression in subsets of patients. The top parts of the figure show, for each patient, the biopsy site, clinical stage, sample type, and dataset. The bottom part shows for each patient whether the sample is enriched for the neuroendocrine (NE) and antigen-presenting-machinery (APM) signatures. (C and D) Relationship between the listed ADC antibody targets and the NMF subgroups (NMFu and NMFr denote the NMFs of samples from untreated and relapsed patients, respectively. The blue-shaded areas include samples with low gene expression (log2(FPKM+1) <3), and the pink-shaded areas include samples with high expression (log2(FPKM+1) >5). (E and F) Expression of drug targets/biomarkers in the different NMF subgroups. (G and H) Expression of drug efflux transporters. (I and J) Expression of major pro-apoptotic genes in the different NMF subgroups. Statistical comparisons were performed by Student’s *t* test. ∗∗∗∗ indicates *p* < 0.001; ∗∗*p* < 0.01; and ∗*p* < 0.05.

SCLC TumorMiner is designed to assess prognosis biomarkers associated with gene expression, mutations, clinical metadata, and NMF signatures. The default display on the website is for *SLFN11* (Figure 5A). *SLFN11*’s wide range of expression in SCLC samples (Figures 6E and 6F) and SCLC cell lines,^4^ as well as its predictive value for predicting response to chemotherapeutic agents targeting DNA replication^28^^,^^29^ (see next section), qualifies it as a quantitative biomarker. Figure 5A shows that high *SLFN11* expression in the untreated TU dataset is associated with improved survival. This observation agrees with a recent publication demonstrating preferential response of *SLFN11*-expressing cells to the DNA-targeted chemotherapies used to treat SCLC.^30^

SCLC TumorMiner also allows multivariable analyses by combining different genes or signatures. Figures 5B and 5C show the predictive value of combining the expression of *SEZ6*, *SRSF1* (encoding the Serine and Arginine Rich Splicing Factor 1),^26^ with the proliferation marker *MKI67* in the 150 untreated patients (HR 2 groups = 1.7; *p* value = 0.016). In the multivariable Cox analysis, age, sex, and *SEZ6* expression were independent predictors of survival, with older age, male sex, and high *SEZ6* associated with increased hazard. *SLFN11* showed a borderline protective effect, while tumor stage and other genes (*MKI67* and *SRFS1*) were not significant after adjustment. These results suggest that *SEZ6* provides prognostic value beyond conventional clinical factors and may improve risk stratification. Additional predictive biomarkers can be searched using the Lasso analysis option (highlighted in yellow at the bottom left in Figure 5A).

The prognostic value of each of the eight NMF gene signatures can also be explored in the “Prognosis Biomarkers” tool of SCLC TumorMiner. Figure 5D shows that at diagnosis before treatment, patients classified as NMF1u appear to have a better prognosis than patients in the MMF2u subgroup. This appears different for patients at relapse, where the NMF3r group appears to be at higher risk (Figure 5D).

The prognostic value of each of the eight gene signatures can also be explored in SCLC TumorMiner. Figure 5E shows the good prognostic value of the APM, immune, and stroma signatures, while male patients tend to have poor survival in the untreated datasets. This observation further indicates that tumors with high immune and stroma infiltration respond better to treatments.

None of the NAPY genes show significant predictive value, even in the untreated patients, despite their broad differential expression (Figure 1). Similarly, *MYC*, *MYCL*, *REST,* and NOTCH do not show significant predictive value. On the other hand, consistent with a recent study reporting that high expression of *SRSF1* is linked with poor survival in patients with SCLC,^26^ we observe that high expression of *SRSF1* is a risk factor for the untreated patients (Figure 5F). Single-gene analyses also confirm the poor prognostic value of *SEZ6* in the untreated patient datasets and the good prognostic value of high *SFLN11* expression in the patients at relapse (Figure 5F).

### Predictive biomarkers for cell surface targets

Thirteen Antibody Drug Conjugates (ADCs) have been approved by the FDA in the past 6 years. Datroway® (Datopotamab Deruxtecan), the most recent (January 2025), delivers the *TOP1* inhibitor Deruxtecan to triple-negative breast cancers (TNBC) expressing *TROP2* (encoded by *TACSTD2*). Notably, the other *TROP2*-targeted *TOP1* ADC, Trodelvy® (Sacituzumab Govitecan), has been granted Breakthrough Therapy Designation for patients with ES-SCLC.^31^ CAR-T cells and bi- and tri-specific T-cell engagers are also in active development, with the recent approval of the T-cell engager Imdelltra® (Tarlatamab-dlle) for ES-SCLC.^32^ However, *DLL3* or *TROP2* are not validated companion diagnostics (CDx) markers for patient selection and, to our knowledge, RNA-seq has not been fully explored in clinical trial analyses.

Figures 6A and 6B display the expression of selected clinically relevant cell surface target genes (Table S9). As shown in Figures 6A and 6C, *DLL3* shows a broad range of expression in the untreated patient samples, with the highest levels in the NE samples matching the NMF1u and NMF2u groups (Figure 6C). Notably, in the samples of patients at relapse, *DLL3* expression is at least 3-fold lower and lacks clear discrimination among the NMF groups (compare Figures 6C and 6D), consistent with the reduced *ASCL1* expression at relapse (Figure 1A). These results suggest the potential of measuring *DLL3* transcripts as a CDx in front-line SCLC.

*TROP2* (encoded by *TACSTD2*) appears generally low at relapse (Figure 6B), while in the untreated patients, its expression is high in samples with low NE score and high APM score (Figure 6A). Accordingly, *TROP2* expression matches the NMF3u (*POU2F3*-endriched) and NMF4u (Immune-enriched) clusters (Figure 6).

Figure 6 (upper A-D) displays the expression of additional cell surface targets for ADCs that are in clinical trials (Table S9). (delta-like non-canonical notch ligand 1) is highly expressed in untreated samples with NE signature (Figure 6A) and less in tumors (Figures 6A–6D). NMF analyses show high *DLK1* expression in the NMF1u and NMF2u and in approximately 50% of the NMF4u group (Inflammatory and Immune) (Figure 6C). These results support the ongoing clinical trial testing a *DLK1*-ADC in NE tumors.^33^

Two of the carcinogenic embryonic antigens (encoded by *CEACAM5* and *CEACAM6*), which are targeted with ADCs in clinical trials for colon, gastric cancers, and NSCLC (Table S9), are highly expressed in the samples classified as NMF1u and NMF2r (Figures 6A–6D). These results suggest the potential of *CEACAM5/6*-targeted ADCs in SCLC and the potential of measuring CEA in the blood of SCLC patients as a CDx biomarker.^34^^,^^35^

*CLDN18* (coding for Claudin 18), an ADC target for pancreatic cancer (Table S9) is highly expressed in a few untreated patients; yet, not in relapsed patients (Figures 6A and 6B). As discussed above, *SEZ6*, a downstream target of *ASCL1,*^36^ is highly expressed across untreated and relapse samples matching the NE signature, with only partial overlap with *DLL3* expression (Figures 6A–6D). Finally, *B7H3* (encoded by *CD276* and belonging to the immunoglobulin superfamily) is expressed in a broad range of samples (Figures 6A–6D). Together, these results validate the potential of *DLL3-, SEZ6-, DLK1-, CEACAM-, CLDN18* and *B7H3*-targeted ADCs in SCLC.^37^

### Predictive pharmacodynamic (PD) biomarkers related to the ADC payloads

The bottom panels of Figure 6 summarize the expression of known intracellular drug targets (Figures 6E and 6F), drug efflux transporters (Figures 6G and 6H), and proapoptotic genes (Figures 6I and 6J).

*SLFN11* (Schlafen 11) shows a broad expression range (over 8-fold) in both the untreated and relapsed patient samples, in agreement with *SLFN11’s* wide distribution across the SCLC cell lines irrespective of the NAPY subgroups^4^ (https://discover.nci.nih.gov/SclcCellMinerCDB/). Consistent with *SLFN11* being an immune- and interferon-response gene,^28^ the NMF4 inflammatory/immune subgroups tend to show the highest *SLFN11* expression both in the untreated and relapse patient samples (Figures 6E and 6F). The broad range of *SLFN11* expression suggests that further studies are warranted to confirm the relevance of *SLFN11* to predict the response of patients treated with lurbinectedin, topotecan, and *TOP1* inhibitor payload ADCs.^38^^,^^39^^,^^40^^,^^41^^,^^42^

Since *TOP1* is the primary cellular target of topotecan, irinotecan, and *TOP1*-ADCs, and *TOP2 is* the target of etoposide, we looked at *TOP1* and *TOP2* expression (Figures 6E and 6F). Both are highly expressed in the samples from untreated and relapsed patients, as expected for housekeeping genes. Thus, *TOP1* and *TOP2* levels are irrelevant as predictive biomarkers.

Lack of O-6-Methylguanine-DNA Methyl-Transferase (*MGMT*) predicts the activity of DNA alkylating agents, foremost among them temozolomide,^43^ which is occasionally used in SCLC.^44^ Figure 6 (right panels E and F) shows low expression of *MGMT* in some samples, suggesting the potential of testing *MGMT* expression for temozolomide treatments.

Drug efflux transporters of the ABC family are a major cause of resistance to tubulin inhibitors (vinca and auristatin derivatives) that are commonly used as ADC payloads. Yet, measuring ABC transporters is not done in clinical routine. Figures 6G and 6H display the expression of the main ABC transporter genes. *ABCB1*, which encodes P-glycoprotein (PgP), has notably low expression both in the untreated and relapse samples, while *ABCC2* and *ABCC3* show significant overexpression in the NMF3r cluster (Figure 6H). These results suggest that measuring ABC transporters should be considered for ADCs with anti-tubulin payloads.

Because broad resistance to chemotherapy is dependent on reduced apoptosis, we explored apoptotic genes. Figures 6I, 6J, and S9C show global suppression of proapoptotic gene expression in relapsed patients, which may account, in part, for the overall resistance of relapsed patients to chemotherapy.

### Analysis of individual patients using the “MyPatient” tab of SCLC TumorMiner

**Figure 7:**
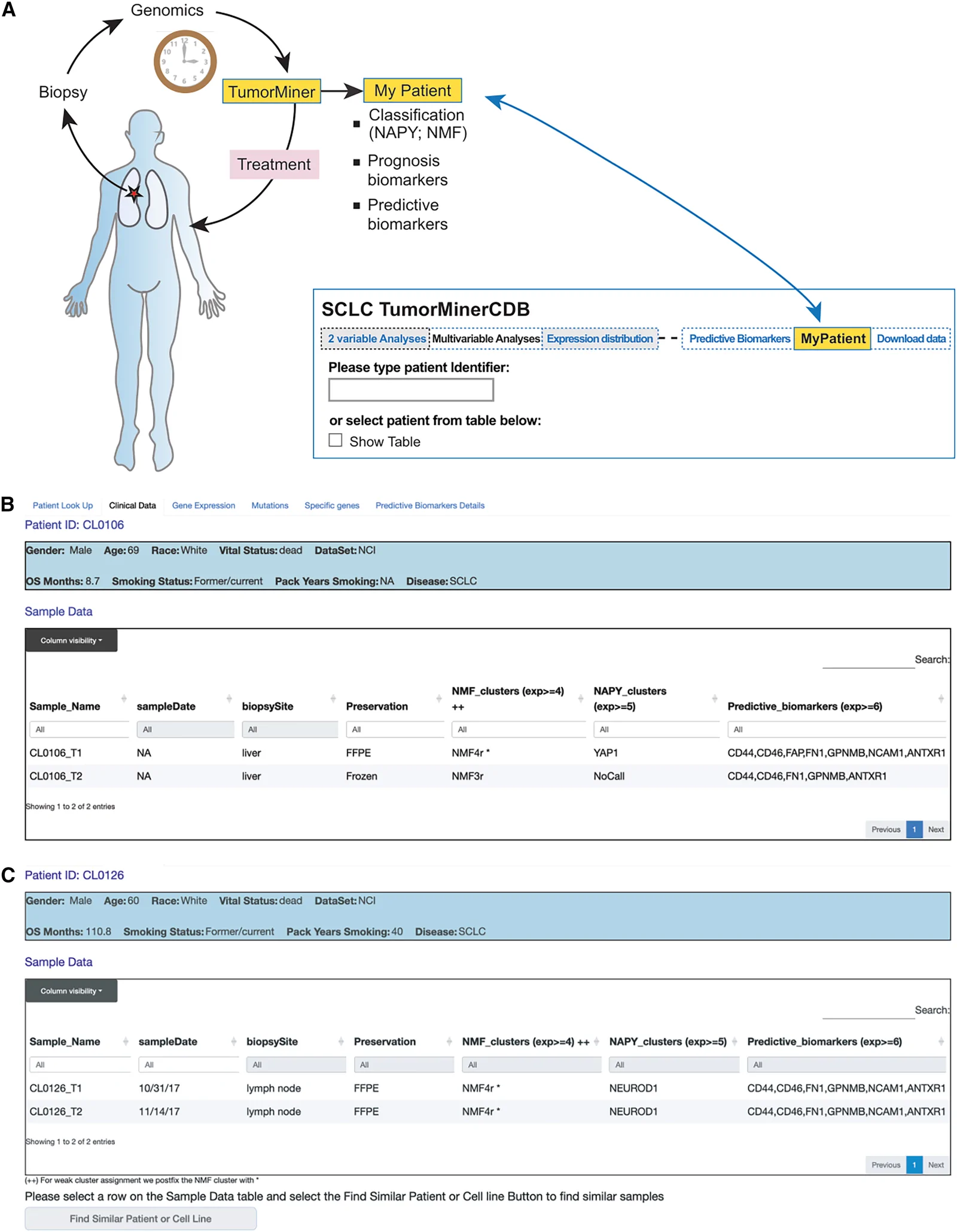
MyPatient module of SCLC TumorMiner (A) Proposed use of the “MyPatient” module. Genomic analyses are performed immediately after biopsy and uploaded into Tumor_Miner to provide results guiding patient treatment, as genomic classification, prognostic, and predictive biomarkers are automatically displayed using the “MyPatient” tab. (B) Snapshot of the “MyPatient” tab in SCLC TumorMiner. Users are prompted to enter a “patient identifier” or select a patient from the available table. Here, the patient has two samples from liver biopsies: one frozen and the other from FFPE. (C) Snapshot of the “MyPatient” tab for patient “CL0126” who has two lymph node biopsy samples taken at a 2-week interval.

Going forward, we propose SCLC TumorMiner as a prototype clinical interface for classifying tumors and for predicting risk factors and rational therapies (Figure 7A). This feature is included in the “My patient” tab of SCLC TumorMiner (Annotation #7 at the top of Figure 1A).

As discussed above (Figure 1B), all the untreated patient samples can be assigned to at least one of the NAPY groups based on the high expression cut-off of log2(FPKM+1) > 5 defined above. Therefore, we have included the NAPY classification to effectively characterize the samples from untreated patients in the “My patient” tab of SCLC TumorMiner. The situation is different for the relapse SCLC samples. Indeed, based on the expression cut-off of log2(FPKM+1) > 5, less than half of the samples (44%) can be assigned to the NAPY groups, with the YAP1 group (SCLC-Y) being prominent (22%) (Figure 1B).

Faced with the limitation of the NAPY classification for the relapsed patient samples and the positive discriminatory value of the NMF classification (Figures 3 and S4), we developed an algorithm to classify the relapse samples based on NMF. Figure S7 shows the average expression scores for the NMFr clusters of the 50 samples from the NCI relapsed patients. Based on this plot, we chose a threshold of 4 to assign samples to each NMFr cluster. This approach enables the computation of mean NMF scores for individual samples. On the website, we implemented this approach to automatically score individual samples in the “MyPatient” tab of SCLC TumorMiner. Upon typing a patient identifier (code) (Figure 7B), SCLC-TumorMiner displays both NAPY and NMF classifiers for cataloging individual patient samples (Figures 7B and 7C).

The tab “My Patient” also displays predictive biomarkers for therapies (Figures 7B and 7C). This is done by the TumorMiner software, which queries a dataset listing 63 cell surface targets for ADCs currently FDA-approved and in clinical development (Table S9). Our website displays the cell surface targets expressed above a threshold of 6 (log2(FPKM+1)). For example, in Figure 7C, the displayed patient tumor sample overexpresses *NCAM1* and *FN1*, implying that the patient could be a candidate for ADCs comprising *NCAM1* or *FN1* antibodies.

These results highlight the translational potential of the SCLC TumorMiner platform for individualized patient care. While traditional NAPY classification remains useful for untreated tumors, its limited applicability to relapsed samples underscores the importance of alternative classifiers. By implementing NMF-based transcriptomic classifiers and integrating biomarker-based predictions for targeted therapies, the “MyPatient” module can provide real-time, personalized tumor profiling. Our goal is to facilitate not only tumor subtype identification but also actionable therapeutics, bridging molecular characterization with clinical decision-making in SCLC. As the platform evolves with more comprehensive datasets, it holds promise for guiding precision medicine strategies in both newly diagnosed and treatment-refractory patients.

## Discussion

Mining cancer patient clinical and genomics data are critical for understanding a disease, discovering biomarkers, predicting outcomes, optimizing treatments, and ultimately shifting patient care to a logic-based and flexible paradigm. During the last decade, tools have emerged such as cBioPortal (http://cbioportal.org)^45^ or Xena (https://xena.ucsc.edu),^46^ GEPIA2 (http://gepia.cancer-pku.cn/),^47^ UALCAN (https://ualcan.path.uab.edu/)^48^ or Survsig (https://survsig.hcemm.eu/), offering different functions for genomic data visualization and analyses.

Here we introduce “SCLC TumorMiner”, a web application for SCLC patient genomics. Its architecture and displays are constructed such as SCLC-CellMinerCDB (https://discover.nci.nih.gov/SclcCellMinerCDB), which enables users to compare tumor samples with a broad collection of SCLC cell lines.^4^^,^^49^ SCLC TumorMiner complements existing tools for mining SCLC patient clinical and genomics information, such as cBioPortal, Xena, or Survsig. It is also meant to improve access and interpretation for physicians and their patients. SCLC TumorMiner has its own specific datasets and unique functionalities to build predictive models and generate prognostic gene sets. It is the first tool to enable comparison between a patient sample and other patient samples or cell lines. Table S10 showcases the distinctive values of SCLC TumorMiner with other tools.

SCLC TumorMiner (https://discover.nci.nih.gov/SclcTumorMinerCDB/) has a battery of unique tools (Figure 1A) allowing real-time display of omics data for individual genes and individual patient samples (*2-Variable Analyses*, *Prognosis Biomarkers* and *Predictive Biomarkers*) or for multiple genes to explore molecular networks (*Multivariable Analyses* and *Compare Patterns* tools) (Figure 1A). It integrates 8 pathway *Signatures* relevant to SCLC biology (NE, RepStress, CES, APM, Immune, EMT, Stroma, Y-Chrom), 50 Hallmark signatures, and 63 cell surface targets relevant to the current development of antibody drug conjugates (ADCs), Bispecific T-cell engagers (BiTEs), and CAR-T cell therapies. Data can be downloaded from the SCLC TumorMiner website for further analyses, as demonstrated in this report by the different plots comparing gene expression in untreated and treated patients and in samples from different NMF groups.

A biologically relevant feature of TumorMiner is its ability to explore the logic of molecular networks/pathways based on the premise that individual components of molecular pathways are quantitatively linked and significantly correlated.^4^ Figures 2 and S2 demonstrate that each of the NAPY genes has its own network (genomic identity) across tumor samples and that these networks are highly conserved across patient samples and cell line models (Figure S2C), implying their robustness. A notable finding is the interconnection between the Notch, the *YAP/TAZ* and the *ASCL1*-NE pathways whereby expression of the *YAP/TAZ* pathway is tightly linked to activation of the Notch pathway; and vice-versa that activation of the NE (*ASCL1-SEZ6*) pathway is associated with expression of the Notch ligands (*DLL3, DLK1, DLL1, DLL4* and *JAG2*) and negatively correlated with the Notch receptors and their transcription effector *REST* (Figure 2F). These results suggest feedback loops involving the non-NE pathways including *YAP/TAZ* and the Notch receptors, and the NE pathway including *ASCL1* and the Notch ligands.^21^^,^^22^ Whether and how *YAP/TAZ* functions upstream of the Notch transcription factor *REST* remains to be established.

Cancer cell plasticity and heterogeneity are two outstanding characteristics of SCLC.^50^^,^^51^^,^^52^^,^^53^^,^^54^^,^^55^^,^^56^ This is exemplified by the fact that at relapse, patients become resistant to chemotherapy after initially responding dramatically. Accordingly, SCLC TumorMiner reveals key genomic differences between the samples of untreated and relapsing patients. One such difference is the loss of the bimodal expression of *NEUROD1, ASCL1,* and *POU2F3* and the upregulation of *YAP1* at relapse (Figure 1). This could have, at least two not mutually exclusive explanations: 1/differentiated cancer cells expressing *ASCL1* (NE cells) or *POU2F3* (Tuft-ionocyte-like cells) are eliminated by the 1^st^ line of chemotherapy (Figures 1A and S1B), leaving behind less differentiated resistant cells; 2/chemotherapy is less effective toward the less differentiated and more plastic tumor cells with basal cell characteristics,^55^ which remain in the tumor and become prominent at relapse.

Another notable difference between untreated tumors and tumors at relapse is the significant downregulation of *MYCL* at relapse (Figure 2G). This reduction is likely consequential to the fact that *ASCL1* is a transcriptional driver of *MYCL*^57^ (as visualized in Figure 2B)^57^ and that *ASCL1* expression is significantly suppressed in relapsed tumors (Figures 1A and 1B).

With respect to the chemoresistance of relapsed tumors, it is likely multifactorial. Indeed, while the expression of *SLFN11* and some of the proapoptotic genes (*BAD, BAX, BID, NOXA* and *PUMA*) is high in chemotherapy-naïve patient samples, it is markedly reduced in relapsed tumors (Figure 6), suggesting that the initial response of patients to various chemotherapies is driven, in part by pro-apoptotic genes and *SLFN11*. Conversely, resistance may involve downregulation of *SLFN11* and the cell death pathways. Whether these pathways are epigenetically suppressed in relapsed tumors and could be reactivated by *HDAC* and *EZH2* inhibitors remains to be tested.^10^^,^^39^^,^^58^

Bispecific antibodies T-cell engagers (BiTEs), antibody-drug conjugates (ADCs), and CAR-T are in full development. Regarding the therapies targeting *DLL3*, Tarlatamab (Imdelltra®) and Zocilurtatug Pelitecan (Zl-1310) (NCT06179069), expression of *DLL3* is generally lower at relapse than in untreated patients, consistent with *DLL3* being a direct transcriptional target of *ASCL1*, which is significantly suppressed at relapse (Figures 6 and S9).^4^^,^^57^ This may justify moving Tarlatamab and Z-1310 to 1^st^ line therapy. Also, since *DLL3* IHC analyses have until now failed to predict responses,^59^ using RNA-seq could identify responders (approximately 40%) to Tarlatamab^32^^,^^59^ and Z-1310 (NCT06179069), thereby avoiding unnecessary toxicity and cost. Surprisingly, expression of *TROP2* (encoded by *TACSTD2*) is generally low in both untreated and relapse patient samples. This raises questions about the potential of Trodelvy® (Sacituzumab Govitecan) or Datroway® (Datopotamab deruxtecan) in patients with SCLC irrespective of their disease stage.

Based on our observations, *SEZ6* appears promising for two of the ADCs in clinical development: ABBV-011 and ABBV-706 (Table S9). Yet, such as *DLL3, SEZ6* is a downstream transcriptional target of *ASCL1*^36^^,^^37^ (Figure 2) and is therefore likely to be more effectively targeted in untreated patients. By contrast, *B7H3* (encoded by *CD276*), the target of two ADCs in advanced clinical trials for ES-SCLC: Risvutatug Rezetecan (GSK5764227) and Ifinatamab Deruxtecan (DS-7300) (Table S9), appears highly expressed in a significant fraction of samples from relapsed patients in SCLC TumorMiner. Our systematic screening for other cell surface targets reveals that CEA (carcinoembryonic antigen), which is encoded by *CEACAM5* and *CEACAM6*, is highly expressed in multiple samples from relapsed patients (Figure 6), suggesting the potential of CEA-ADCs.^60^^,^^61^^,^^62^ An advantage of targeting CEA is that, in addition to measuring *CEACAAM5/6* transcripts and by IHC in tumor samples, clinical trials could include CEA blood samples.^63^^,^^64^ We also found that *TRPM5*, which encodes a sodium channel transporter, is selectively expressed in the *POU2F3*-expressing tumors and that glial cell line-derived neurotrophic factor receptor alpha (*GFRA1*) and *SEMA6A* (encoding one of the semaphorins)^14^ are specifically expressed in *NEUROD1*-expressing SCLC (Figure 2), suggesting they might be considered as cell surface targets for SCLC. These analyses demonstrate the potential use of SCLC TumorMiner for discovering therapeutic targets for ADCs, T-Cell Engagers (BiTEs), and CAR-T.

As we move toward precision medicine, multivariable analyses will be needed. For instance, for a given cell surface target, clinicians may have to choose between an ADC with a *TOP1* or a tubulin payload or a T-cell engager (BiTE). To illustrate this point, one could propose guiding payload choice with at least two biomarkers: *SLFN11* and ABC drug efflux transporters. If the tumor expresses high *SLFN11* and drug efflux transporters, TOP1 inhibitors will likely be superior to a tubulin inhibitor ADC, and conversely, a tubulin inhibitor ADC payload would be logical for tumors with low *SLFN11* and ABC transporter expression. For immune checkpoint inhibitors and BiTEs, one could consider testing the predictive value of high APM and Immune signatures, which would reflect the presence of immune cells in the tumor, or tumor intrinsic features such as high APM or *NOTCH1* expression.^65^^,^^66^

Ultimately, we speculate that “*MyPatient*” could be developed for clinical practice, as bulk RNA-seq and DNA-seq can routinely be performed. Sequencing results and clinical parameters could be loaded into SCLC TumorMiner. Additionally, the Tumor_Miner architecture and interface could be readily expanded to integrate protein expression (proteomics) and epigenetic (DNA methylome) parameters as we have already done in our CellMiner websites (https://discover.nci.nih.gov/). “My_Patient” could provide a medical interface displaying a patient’s genomic parameters, dictating which therapy would be most appropriate and predicting risk factors. Finally, one could anticipate future integration of natural-language query interfaces or Chatbot to enhance user accessibility.

### Limitations of the study

A limitation of the current study is the technical and clinical heterogeneity of the cohorts. RNA-seq data were generated from both fresh-frozen and FFPE samples, with untreated cohorts primarily derived from frozen tissues and the relapse NCI cohort including both frozen and FFPE samples. Analysis of the NCI samples, including 8 frozen biopsy samples and 42 FFPE samples, shows that preservation methods only explain 2.9% of the variance, which is quite low, especially compared to the biopsy site, which explains 10.8% of the variance. Hierarchical clustering analysis of the 204 samples from the 3 datasets (UoC, TU, and NCI) also shows a clear separation between NCI (relapse) and the other samples (Untreated). The NCI relapse samples are mostly FFPE, whereas the untreated samples are all frozen. We cannot remove the effect of the preservation because it is confounded with sample type (relapsed vs. untreated). Thus, no batch effect was applied for all 3 datasets, and we believe that bulk RNA-seq of patient samples can give a valuable estimate of gene expression for highly expressed genes [log2(FPKM+1) > 5]. Adding more samples will increase the statistical power and robustness of SCLC TumorMiner. However, a challenge for cross-database analyses is to access large and well-annotated datasets. Material transfer agreements commonly restrict the sharing of FASTQ files and associated clinical data across institutions. “MyPatient” module only provides a prototype, and users will have the option to integrate our TumorMiner platform into Electronic Medical Record (EMR) systems to provide “real-time” evaluation of tumor omics for precision medicine.

## Resource availability

### Lead contact

Further information and requests for reagents may be directed to and will be fulfilled by Lead Contact Yves Pommier (pommier@nih.gov).

### Materials availability

This study did not generate new unique reagents.

### Data and code availability

• The data used for the analysis can be downloaded from the website at https://discover.nci.nih.gov/SclcTumorMinerCDB/or obtained from Zenodo at https://doi.org/10.5281/zenodo.18343141. The clinical information for all three datasets is also provided in Supplementary Data.
• The website code is publicly available in GitHub at https://github.com/cbiit/SclcTumorMinerCDB.
• Any additional information or script required to reanalyze the data reported in this paper is available from the lead contact upon request.

## Acknowledgments

This research was supported [in part] by the Intramural Research Program of the National Institutes of Health (NIH). The contributions of the NIH author(s) are considered Works of the United States Government. The findings and conclusions presented in this paper are those of the author(s) and do not necessarily reflect the views of the NIH or the U.S. Department of Health and Human Services. This work is funded by the Intramural Program of the National Cancer Institute, NIH (Z01-BC 006150). In addition, this work is also supported by the Division of Intramural Research (DIR) of the National Library of Medicine (NLM) (ZIALM240126). We thank the NCI CCR Collaborative Bioinformatics resource (CCBR) team for their help in running the RNA-seq and Exome-seq pipelines.

## Author contributions

Y.P. supervised the study. Y.P., F.E., and Y.A. devised the concept. Y.A., D.T., A.T., A.L., J.D.R, F.A., K.A., and M.R. performed data collection and clinical annotations. Y.P., F.E., A.D., S.V., D.T., Y.A., A.L., R.S., N.R., A.T., C.R., A.T., and W.R performed data analysis. F.E., J.W. and A.T. built the website. Y.P., F.E., A.D., A.L., A.T., M.A., N.R., C.R., and W.R. wrote and critically reviewed the manuscript. All authors reviewed the results and approved the final version of the manuscript.

## Declaration of interests

The authors declare no competing interests.

## STAR★Methods

### Key resources table

**Table:** 

REAGENT or RESOURCE	SOURCE	IDENTIFIER
**Deposited data**		

NIH NCI – Patient Data	Lissa et al.^52^	dbGAP: phs002541.v1.p1
SCLC tumor transcriptomic data	George et al.^2^	EGA:EGAS00001000925
Tongji SCLC data	Liu et al.^9^	https://ngdc.cncb.ac.cn/gsa-human/browse/HRA003419
Impower133 tumor transcriptomic data	Gay et al.^5^	EGA:EGAS00001004888
Wagner dataset	Wagner et al.^27^	dbGAP: phs001049.v1.p1
SCLC tumor transcriptomic data	Jiang et al.^26^	GEO:GSE60052

**Software and algorithms**		

R version 4.5.1	CRAN	www.r-project.org
Bioconductor	Bioconductor	www.bioconductor.org
RNASeq pipeline	NCI CCBR	https://github.com/skchronicles/RNA-seek
Mutation pipeline	NCI CCBR	https://github.com/mtandon09/CCBR_GATK4_Exome_Seq_Pipeline
GSVA	Bioconductor	version 2.4.4
fGSEA	Bioconductor	version 4.2.3
NMF	CRAN	version 0.28
Survival	CRAN	version 3.8.3
GraphPad Prism	GraphPad	N/A
Analysis scripts	This paper	https://zenodo.org/records/18343141
SCLC TumorMinerCDB	This paper	https://github.com/cbiit/SclcTumorMinerCDB

### Experimental model and study participant details

Patient clinical and genomics data were collected from our DTB clinic at the Center for Cancer Research, NCI-NIH, the University of Cologne (UoC) and Tongji university (TU). Our genomics patient data currently includes a total of 204 samples from 189 patients with RNA-seq and/or whole-exome sequencing (WES) samples. The NIH samples were obtained from patients enrolled in IRB-approved protocols (NCT01851395, NCT02146170, NCT02484404, and NCT02487095) and had consented to the analysis of their tumors. Clinical data from NIH and collaborators were gathered and manually curated by a team of DTB physicians. They integrate patient demographics, survival, treatment with response, and sample information, including sample type, biopsy site, tumor stage and pathology indicators (Table S1). NCI patient samples were correlated with treatment response, and for each patient we selected the first drug(s) or treatment(s) post-sampling as well as its response class (CR: complete response, PR: partial response, PD: progressive disease or SD: stable disease). Due to the current limited number of treatments, we decided that the response will be of value 1 (good responder: PR, CR) or 0 (bad responder: SD, PD).

### Method details

#### Genomic content and infrastructure

All genomics patient data were processed with our on-site data-handling pipelines using the raw data. We generated 204 RNA-seq and 114 WES normalized data. Treatment responses were available for 14 patients. Table S11 shows details by disease, datasets and experiments.

We also computed for each sample gene expression Hallmark enrichment and genomics signatures scores such as the NE, RepStress, EMT, APM, the level of stroma and immune infiltrate contamination or a Y chromosome signature score. Table S12 presents the list of signatures as well as the phenotypic data queryable on SCLC TumorMiner.

SCLC TumorMiner has similar design as the CellMinerCDB tool and websites (https://discover.nci.nih.gov/) dedicated to mining pharmaco-genomics data across broad ranges of cancer cell line sets and databases. SCLC TumorMiner is implemented as a shiny application and hosted using Posit Connect on CBIIT servers at https://discover.nci.nih.gov/SclcTumorMinerCDB/.

#### RNA sequencing and batch assessment

The NIH/NCI (relapse) cohort was composed predominantly of FFPE specimens (*n* = 42), with a limited number of frozen samples (*n* = 8) using the Illumina access protocol, whereas the Universities of Cologne (UoC) and Tongji university (TU) were from Frozen using PolyA protocol. All the SCLC samples were processed by running the NCI CCBR RNAseq pipeline (https://github.com/skchronicles/RNA-seek.git). STAR (2.7.6a) was run to map reads to hg38 reference genome (release 36). Then RSEM was used to have gene expression values in log2(FPKM+1). For the NCI dataset, we checked whether the way the samples were preserved—Frozen or FFPE—affects gene expression, along with other factors like biopsy site, sex, and age. Analysis of the NCI samples including 8 frozen biopsy samples and 42 FFPE samples shows that preservation methods only explain 2.9% of the variance, which is quite low, especially compared to the biopsy site, which explains 10.8%. All untreated samples are frozen. Because the preservation method is confounded with sample type (relapse vs. untreated), its effect cannot be disentangled (Figure S8). Thus, no batch effect was applied for all 3 datasets.

#### Mutations

Somatic mutations were based on paired tumor-normal samples and ran with the CCBR exome sequencing pipeline (https://github.com/mtandon09/CCBR_GATK4_Exome_Seq_Pipeline). Briefly, BWA MEM (version 0.7.17) was run to map reads to the hg38 reference genome. Then Mutect2 in GATK 4.2 was used to call the variants.

Across 98 Tongji University paired normal and tumor exomes, the median of the mean target depth was 161.57× (IQR 139.32–189.27×), and a median of 97.1% (IQR 96.2–97.7%) of target bases were covered at ≥30×. For NCI, across 16 paired normal and tumor exomes, the median of the mean target depth was 97.75× (IQR 62.9–143.5×), and a median of 90.9% (IQR 79.9–95.42%) of target bases were covered at ≥30×. Sample coverage details are provided in Tables S13 and S14.

Variants with 6 or more total filtered reads were selected for display. We used Annovar (hg38 version) to annotate the mutations, including their predicted effect on amino acid sequence and whether they are predicted to be deleterious, as well as their prevalence in the ExAC normal population. A mutation was called deleterious if its predicted effect on the RNA sequence was frameshift, nonsense, splice-site, or a missense mutation with a SIFT score <0.05 or PolyPhen2 HDIV score>=0.85. We selected all deleterious variants for the mutation gene-level score.

The fraction of alternate alleles (the mutation ratio) was used as the probability that any cell has that mutation. Furthermore, we assumed that the probability that any mutation is present in a gene is independent of the probability of any other mutation on that gene. Given those assumptions, we calculate the overall mutation probability for that gene in any cell as:

1−(1−p1)∗(1−p2)∗...∗(1−pn)

where p1, p2, …, pn are the mutation ratios for each individual mutation on that gene. 1-pj is the probability that jth mutation is not present for a particular cell. Thus, the product is the probability that none of the mutations are present and 1 minus the product is the probability that at least one mutation is present. The expression gives a number that is between 0 and 1, which we multiply by 100 to get the mutation score for that gene.

#### Tumor mutation burden (TMB)

The TMB was computed using the R package MAFtools^67^ based on Mutect2 somatic variants that have variant allele frequency (VAF) ≥ 5%.

#### Genomic data for survival

If a patient has multiple samples, we select the primary tumor sample (if available) for survival analysis or we choose one based on biopsy site. The list of selected samples is downloadable from the website.

### Quantification and statistical analysis

#### Unsupervised clustering approach

We implemented unsupervised clustering based on Non-Negative Matrix Factorization (NMF) algorithm using log-transformed normalized RNA-seq data to classify SCLC patients into molecular subtypes. NMF uses dimensionality reduction approach in which it decomposes the gene expression matrix X (n × m), into the product of two lower-dimension non-negative matrices (See Equation below), where n represents number of patients and m is number of genes.

X≈WH

with X = n × m matrix, W = n × k matrix, and H = m × k, where k is a latent factor; optimal number of factors (k) was determined based on matrix stability using cophenetic correlation coefficient.

We selected only protein-coding genes for both untreated and relapse SCLC samples, then we selected the top 5000 highly variable genes for both datasets using a non-parametric approach, Median Absolute Deviation (MAD) using “mad” function in R (version 4.2.3). After which, we performed consensus NMF clustering evaluating values of k ranging from 2 to 8, based on cophenetic correlation coefficient. k = 4 provided most stable and robust clusters for both untreated and relapse SCLC datasets (Figure S4). Clustering was performed using “NMF” library in R (version 4.2.3).

#### Pairwise differential expression analysis

After defining the NMF subgroups, differential gene analyses were performed using the “Limma” package in R (version 4.2.3). To determine the up or down-regulated genes across the NMF- subgroups, we performed pairwise comparisons for untreated and relapse SCLC separately. This included all possible combinations of comparisons: NMF1 vs. NMF2, NMF1 vs. NMF3, NMF3 vs. NMF4, NMF2 vs. NMF3, NMF2 vs. NMF4, NMF3 vs. NMF4. In addition, each subgroup was compared with the aggregated sum of other subgroups, for instance: NMF1 vs. Others (NMF2+NMF3+NMF4), NMF2 vs. Others (NMF1+NMF3+NMF4), NMF3 vs. Others (NMF1+NMF2+NMF4) and NMF4 vs. Others (NMF1+NMF2+NMF3).

Subsequently, we utilized these pairwise comparisons to identify subgroup-specific genes for each NMF cluster for both untreated and relapse samples. Significantly upregulated genes in NMF1 in comparison to NMF2, NMF3 and NMF4 with log_2_FC > 1 and *p*-value <0.05 are listed as NMF1-specific genes. Similarly, this criterion was implemented for the selection of specific genes for the NMF2, NMF3 and NMF4 subgroups. The subgroup-specific genes identified using this approach were used to construct NMF-derived gene expression signatures.

#### Gene set enrichment analysis (GSEA)

To investigate the functional relevance of genes we performed pre-ranked Gene Set Enrichment Analysis (GSEA). From previous analysis, we obtained pairwise comparisons for each NMF groups and used t-statistic parameter, which provided magnitude and direction of differential expression to rank the genes. Ranked genes were then analyzed using the fGSEA R package (version 4.2.3) with REACTOME gene sets [https://www.gsea-msigdb.org/gsea/msigdb/human/collections.jsp#C5]. fGSEA is a fast implementation of the GSEA algorithm providing normalized enrichment scores (NES) and adjusting for multiple testing using permutation-based *p*-values and false discovery rate (FDR).

#### Mypatient NMF cluster assignment

For each patient sample, we calculated the average expression of the four NMF signatures (Figure S7). The assigned NMF cluster corresponds to the signature with the highest average expression, provided that this value is at least 4 (log2(FPKM+1)); otherwise, no cluster is assigned. If the highest average expression differs from the second highest by less than 1, the cluster assignment is considered weak and is labeled accordingly.

#### Statistical methods

Correlations, scatterplots, heatmaps, survival analysis, regression model predictions, and pathway enrichment scores were generated using the R-Project for statistical computing based mostly on the R packages: ggplot2, plotly, heatmaply, survival, glmnet, ggforest, GSVA and GSEABase. These packages are downloadable from the comprehensive R archive network (CRAN) or Bioconductor websites. Statistical comparisons shown in boxplots were performed using Student’s *t* test. Statistical significance was denoted as follows: ∗∗∗∗, *p* < 0.001; ∗∗, *p* < 0.01; and ∗, *p* < 0.05.

## Supplemental information

Document S1. Figures S1–S8

Table S1. Clinical and demographic characteristics of small cell lung cancer patients

Table S2. RB1 depletion per sample

Table S3. NMF untreated results A. Top-5000 Genes, B: NMF1u_vs._others, C: NMF2u_vs._others, D: NMF3u_vs._others, E: NMF4u_vs._others, F: NMF1u_vs._NMF2u, G: NMF1u_vs._NMF3u, H: NMF1u_vs._NMF4u, I: NMF2u_vs._NMF3u, J: NMF2u_vs._NMF4u, K: NMF3u_vs._NMF4u.

Table S4. NMF relapse results A. Top-5000 Genes, B: NMF1r_vs._others, C: NMF2r_vs._others, D: NMF3r_vs._others, E: NMF4r_vs._others, F: NMF1r_vs._NMF2r, G: NMF1r_vs._NMF3r, H: NMF1r_vs._NMF4r, I: NMF2r_vs._NMF3r, J: NMF2r_vs._NMF4r, K: NMF3r_vs._NMF4r.

Table S5. NMF relapse selected genes A. Based on differentially expressed genes, B: Top30.

Table S6. NMF untreated selected genes A. Based on differentially expressed genes, B: Top30.

Table S7. Reactome pathway enrichment results in the untreated cohort A: NMF1u_vs._others, B: NMF2u_vs._others, C: NMF3u_vs._others, D: NMF4u_vs._others.

Table S8. Reactome pathway enrichment results in the relpase cohort A: NMF1r_vs._others, B: NMF2r_vs._others, C: NMF3r_vs._others, D: NMF4r_vs._others.

Table S9. Antibody-drug conjugates (ADCs) A: Drug information. B: List of unique ADC target genes.

Table S10. Comparison of SCLC TumorMiner to similar tools

Table S11. Sample data type summary

Table S12. List of gene signatures

Table S13. Tongyu whole-exome sequencing (WES) samples coverage

Table S14. NCI whole-exome sequencing (WES) samples coverage

## References

[1] Thomas A., Takahashi N., Oplustil O’Connor L., Redon C.E., Mohindroo C., Sciuto L., Pongor L., Schmidt K.T., Steinberg S.M., Aladjem M.I. (2025). Tumor-targeted top1 inhibitor delivery with optimized parp inhibition in advanced solid tumors: a phase i trial of gapped scheduling. Nat. Commun..

[2] George J., Lim J.S., Jang S.J., Cun Y., Ozretić L., Kong G., Leenders F., Lu X., Fernández-Cuesta L., Bosco G. (2015). Comprehensive genomic profiles of small cell lung cancer. Nature.

[3] Rudin C.M., Poirier J.T., Byers L.A., Dive C., Dowlati A., George J., Heymach J.V., Johnson J.E., Lehman J.M., MacPherson D. (2019). Molecular subtypes of small cell lung cancer: a synthesis of human and mouse model data. Nat. Rev. Cancer.

[4] Tlemsani C., Pongor L., Elloumi F., Girard L., Huffman K.E., Roper N., Varma S., Luna A., Rajapakse V.N., Sebastian R. (2020). SCLC-CellMiner: A Resource for Small Cell Lung Cancer Cell Line Genomics and Pharmacology Based on Genomic Signatures. Cell Rep..

[5] Gay C.M., Stewart C.A., Park E.M., Diao L., Groves S.M., Heeke S., Nabet B.Y., Fujimoto J., Solis L.M., Lu W. (2021). Patterns of transcription factor programs and immune pathway activation define four major subtypes of SCLC with distinct therapeutic vulnerabilities. Cancer Cell.

[6] Pacheco J., Bunn P.A. (2019). Advancements in Small-cell Lung Cancer: The Changing Landscape Following IMpower-133. Clin. Lung Cancer.

[7] Paz-Ares L., Dvorkin M., Chen Y., Reinmuth N., Hotta K., Trukhin D., Statsenko G., Hochmair M.J., Özgüroğlu M., Ji J.H. (2019). Durvalumab plus platinum-etoposide versus platinum-etoposide in first-line treatment of extensive-stage small-cell lung cancer (CASPIAN): a randomised, controlled, open-label, phase 3 trial. Lancet.

[8] Nabet B.Y., Hamidi H., Lee M.C., Banchereau R., Morris S., Adler L., Gayevskiy V., Elhossiny A.M., Srivastava M.K., Patil N.S. (2024). Immune heterogeneity in small-cell lung cancer and vulnerability to immune checkpoint blockade. Cancer Cell.

[9] Liu Q., Zhang J., Guo C., Wang M., Wang C., Yan Y., Sun L., Wang D., Zhang L., Yu H. (2024). Proteogenomic characterization of small cell lung cancer identifies biological insights and subtype-specific therapeutic strategies. Cell.

[10] Tang S.W., Thomas A., Murai J., Trepel J.B., Bates S.E., Rajapakse V.N., Pommier Y. (2018). Overcoming Resistance to DNA-Targeted Agents by Epigenetic Activation of Schlafen 11 (SLFN11) Expression with Class I Histone Deacetylase Inhibitors. Clin. Cancer Res..

[11] Pozo K., Kollipara R.K., Kelenis D.P., Rodarte K.E., Ullrich M.S., Zhang X., Minna J.D., Johnson J.E. (2021). ASCL1, NKX2-1, and PROX1 co-regulate subtype-specific genes in small-cell lung cancer. iScience.

[12] Anderson K.R., Torres C.A., Solomon K., Becker T.C., Newgard C.B., Wright C.V., Hagman J., Sussel L. (2009). Cooperative transcriptional regulation of the essential pancreatic islet gene NeuroD1 (beta2) by Nkx2.2 and neurogenin 3. J. Biol. Chem..

[13] Gosmain Y., Marthinet E., Cheyssac C., Guérardel A., Mamin A., Katz L.S., Bouzakri K., Philippe J. (2010). Pax6 controls the expression of critical genes involved in pancreatic alpha cell differentiation and function. J. Biol. Chem..

[14] Mastrantonio R., You H., Tamagnone L. (2021). Semaphorins as emerging clinical biomarkers and therapeutic targets in cancer. Theranostics.

[15] Huang Y.H., Klingbeil O., He X.Y., Wu X.S., Arun G., Lu B., Somerville T.D.D., Milazzo J.P., Wilkinson J.E., Demerdash O.E. (2018). POU2F3 is a master regulator of a tuft cell-like variant of small cell lung cancer. Genes Dev..

[16] Montoro D.T., Haber A.L., Biton M., Vinarsky V., Lin B., Birket S.E., Yuan F., Chen S., Leung H.M., Villoria J. (2018). A revised airway epithelial hierarchy includes CFTR-expressing ionocytes. Nature.

[17] Ireland A.S., Xie D.A., Hawgood S.B., Barbier M.W., Zuo L.Y., Hanna B.E., Lucas-Randolph S., Tyson D.R., Witt B.L., Govindan R. (2025). Basal cell of origin resolves neuroendocrine-tuft lineage plasticity in cancer. Nature.

[18] Wu X.S., He X.Y., Ipsaro J.J., Huang Y.H., Preall J.B., Ng D., Shue Y.T., Sage J., Egeblad M., Joshua-Tor L., Vakoc C.R. (2022). OCA-T1 and OCA-T2 are coactivators of POU2F3 in the tuft cell lineage. Nature.

[19] Abdel Wadood N., Hollenhorst M.I., Elhawy M.I., Zhao N., Englisch C., Evers S.B., Sabachvili M., Maxeiner S., Wyatt A., Herr C. (2025). Tracheal tuft cells release ATP and link innate to adaptive immunity in pneumonia. Nat. Commun..

[20] Baine M.K., Febres-Aldana C.A., Chang J.C., Jungbluth A.A., Sethi S., Antonescu C.R., Travis W.D., Hsieh M.S., Roh M.S., Homer R.J. (2022). POU2F3 in SCLC: Clinicopathologic and Genomic Analysis With a Focus on Its Diagnostic Utility in Neuroendocrine-Low SCLC. J. Thorac. Oncol..

[21] Shue Y.T., Drainas A.P., Li N.Y., Pearsall S.M., Morgan D., Sinnott-Armstrong N., Hipkins S.Q., Coles G.L., Lim J.S., Oro A.E. (2022). A conserved YAP/Notch/REST network controls the neuroendocrine cell fate in the lungs. Nat. Commun..

[22] Kim J.W., Ko J.H., Sage J. (2022). DLL3 regulates Notch signaling in small cell lung cancer. iScience.

[23] Ireland A.S., Micinski A.M., Kastner D.W., Guo B., Wait S.J., Spainhower K.B., Conley C.C., Chen O.S., Guthrie M.R., Soltero D. (2020). MYC Drives Temporal Evolution of Small Cell Lung Cancer Subtypes by Reprogramming Neuroendocrine Fate. Cancer Cell.

[24] Gazdar A.F., Bunn P.A., Minna J.D. (2017). Small-cell lung cancer: what we know, what we need to know and the path forward. Nat. Rev. Cancer.

[25] Gazdar A.F., Carney D.N., Nau M.M., Minna J.D. (1985). Characterization of variant subclasses of cell lines derived from small cell lung cancer having distinctive biochemical, morphological, and growth properties. Cancer Res..

[26] Jiang L., Huang J., Higgs B.W., Hu Z., Xiao Z., Yao X., Conley S., Zhong H., Liu Z., Brohawn P. (2016). Genomic Landscape Survey Identifies SRSF1 as a Key Oncodriver in Small Cell Lung Cancer. PLoS Genet..

[27] Wagner A.H., Devarakonda S., Skidmore Z.L., Krysiak K., Ramu A., Trani L., Kunisaki J., Masood A., Waqar S.N., Spies N.C. (2018). Recurrent WNT pathway alterations are frequent in relapsed small cell lung cancer. Nat. Commun..

[28] Jo U., Pommier Y. (2022). Structural, molecular, and functional insights into Schlafen proteins. Exp. Mol. Med..

[29] Murai J., Thomas A., Miettinen M., Pommier Y. (2019). Schlafen 11 (SLFN11), a restriction factor for replicative stress induced by DNA-targeting anti-cancer therapies. Pharmacol. Ther..

[30] Masuda K., Yoshida T., Motoi N., Shinno Y., Matsumoto Y., Okuma Y., Goto Y., Horinouchi H., Yamamoto N., Watanabe S.-i. (2025). Schlafen 11 Expression in Patients With Small Cell Lung Cancer and Its Association With Clinical Outcomes. Thorac. Cancer.

[31] Dowlati A., Chiang A.C., Cervantes A., Babu S., Hamilton E., Wong S.F., Tazbirkova A., Sullivan I.G., van Marcke C., Italiano A. (2025). Phase 2 Open-Label Study of Sacituzumab Govitecan as Second-Line Therapy in Patients With Extensive-Stage SCLC: Results From TROPiCS-03. J. Thorac. Oncol..

[32] Ahn M.-J., Cho B.C., Felip E., Korantzis I., Ohashi K., Majem M., Juan-Vidal O., Handzhiev S., Izumi H., Lee J.-S. (2023). Tarlatamab for Patients with Previously Treated Small-Cell Lung Cancer. N. Engl. J. Med. Overseas. Ed..

[33] Sun N.Y., Kumar S., Kim Y.S., Varghese D., Mendoza A., Nguyen R., Okada R., Reilly K., Widemann B., Pommier Y. (2025). Identification of DLK1, a Notch ligand, as an immunotherapeutic target and regulator of tumor cell plasticity and chemoresistance in adrenocortical carcinoma. Nat. Commun..

[34] Han K., Chen Y., Sun X., Wen L., Wu Y., Chen S., Wei L., Yu J., Zeng T., Jiang L., Tan L. (2025). Combining serum CDK1 with tumor markers for the diagnosis of small cell lung cancer. Clin. Transl. Oncol..

[35] Muley T., Herth F.J., Heussel C.P., Kriegsmann M., Thomas M., Meister M., Schneider M.A., Wehnl B., Mang A., Holdenrieder S. (2024). Prognostic value of tumor markers ProGRP, NSE and CYFRA 21-1 in patients with small cell lung cancer and chemotherapy-induced remission. Tumour Biol..

[36] Kudoh S., Tenjin Y., Kameyama H., Ichimura T., Yamada T., Matsuo A., Kudo N., Sato Y., Ito T. (2020). Significance of achaete-scute complex homologue 1 (ASCL1) in pulmonary neuroendocrine carcinomas; RNA sequence analyses using small cell lung cancer cells and Ascl1-induced pulmonary neuroendocrine carcinoma cells. Histochem. Cell Biol..

[37] Ajay A., Wang H., Rezvani A., Savari O., Grubb B.J., McColl K.S., Yoon S., Joseph P.L., Kopp S.R., Kresak A.M. (2025). Assessment of targets of antibody drug conjugates in SCLC. npj Precis. Oncol..

[38] Qu S., Fetsch P., Thomas A., Pommier Y., Schrump D.S., Miettinen M.M., Chen H. (2022). Molecular Subtypes of Primary SCLC Tumors and Their Associations With Neuroendocrine and Therapeutic Markers. J. Thorac. Oncol..

[39] Sun F., Das M.S. (2024). Targeting the EZH2-SLFN11 pathway-a lesson in developing molecularly-informed treatments for recurrent small cell lung cancer. Transl. Cancer Res..

[40] Zhang B., Stewart C.A., Wang Q., Cardnell R.J., Rocha P., Fujimoto J., Solis Soto L.M., Wang R., Novegil V., Ansell P. (2022). Dynamic expression of Schlafen 11 (SLFN11) in circulating tumour cells as a liquid biomarker in small cell lung cancer. Br. J. Cancer.

[41] Kundu K., Cardnell R.J., Zhang B., Shen L., Stewart C.A., Ramkumar K., Cargill K.R., Wang J., Gay C.M., Byers L.A. (2021). SLFN11 biomarker status predicts response to lurbinectedin as a single agent and in combination with ATR inhibition in small cell lung cancer. Transl. Lung Cancer Res..

[42] Laroche-Clary A., Chaire V., Valverde V., Derieppe M.A., Italiano A. (2025). Synergistic Effects of ATR Inhibition and Lurbinectedin in Soft-Tissue Sarcomas: The Predictive Role of SLFN11 Expression. Clin. Cancer Res..

[43] Thomas A., Tanaka M., Trepel J., Reinhold W.C., Rajapakse V.N., Pommier Y. (2017). Temozolomide in the Era of Precision Medicine. Cancer Res..

[44] Schultz C.W., Zhang Y., Elmeskini R., Zimmermann A., Fu H., Murai Y., Wangsa D., Kumar S., Takahashi N., Atkinson D. (2023). ATR inhibition augments the efficacy of lurbinectedin in small-cell lung cancer. EMBO Mol. Med..

[45] Gao J., Aksoy B.A., Dogrusoz U., Dresdner G., Gross B., Sumer S.O., Sun Y., Jacobsen A., Sinha R., Larsson E. (2013). Integrative analysis of complex cancer genomics and clinical profiles using the cBioPortal. Sci. Signal..

[46] Goldman M.J., Craft B., Hastie M., Repečka K., McDade F., Kamath A., Banerjee A., Luo Y., Rogers D., Brooks A.N. (2020). Visualizing and interpreting cancer genomics data via the Xena platform. Nat. Biotechnol..

[47] Tang Z., Kang B., Li C., Chen T., Zhang Z. (2019). GEPIA2: an enhanced web server for large-scale expression profiling and interactive analysis. Nucleic Acids Res..

[48] Chandrashekar D.S., Karthikeyan S.K., Korla P.K., Patel H., Shovon A.R., Athar M., Netto G.J., Qin Z.S., Kumar S., Manne U. (2022). UALCAN: An update to the integrated cancer data analysis platform. Neoplasia.

[49] Polley E., Kunkel M., Evans D., Silvers T., Delosh R., Laudeman J., Ogle C., Reinhart R., Selby M., Connelly J. (2016). Small Cell Lung Cancer Screen of Oncology Drugs, Investigational Agents, and Gene and microRNA Expression. J. Natl. Cancer Inst..

[50] Simpson K.L., Rothwell D.G., Blackhall F., Dive C. (2025). Challenges of small cell lung cancer heterogeneity and phenotypic plasticity. Nat. Rev. Cancer.

[51] Chan J.M., Quintanal-Villalonga Á., Gao V.R., Xie Y., Allaj V., Chaudhary O., Masilionis I., Egger J., Chow A., Walle T. (2021). Signatures of plasticity, metastasis, and immunosuppression in an atlas of human small cell lung cancer. Cancer Cell.

[52] Lissa D., Takahashi N., Desai P., Manukyan I., Schultz C.W., Rajapakse V., Velez M.J., Mulford D., Roper N., Nichols S. (2022). Heterogeneity of neuroendocrine transcriptional states in metastatic small cell lung cancers and patient-derived models. Nat. Commun..

[53] Stewart C.A., Gay C.M., Xi Y., Sivajothi S., Sivakamasundari V., Fujimoto J., Bolisetty M., Hartsfield P.M., Balasubramaniyan V., Chalishazar M.D. (2020). Single-cell analyses reveal increased intratumoral heterogeneity after the onset of therapy resistance in small-cell lung cancer. Nat. Cancer.

[54] Zhang W., Girard L., Zhang Y.A., Haruki T., Papari-Zareei M., Stastny V., Ghayee H.K., Pacak K., Oliver T.G., Minna J.D., Gazdar A.F. (2018). Small cell lung cancer tumors and preclinical models display heterogeneity of neuroendocrine phenotypes. Transl. Lung Cancer Res..

[55] Ireland A.S., Hawgood S.B., Xie D.A., Barbier M.W., Lucas-Randolph S., Tyson D.R., Zuo L.Y., Witt B.L., Govindan R., Dowlati A. (2024). Basal cell of origin resolves neuroendocrine–tuft lineage plasticity in cancer. bioRxiv.

[56] Qi J., Zhang J., Liu N., Zhao L., Xu B. (2022). Prognostic Implications of Molecular Subtypes in Primary Small Cell Lung Cancer and Their Correlation With Cancer Immunity. Front. Oncol..

[57] Borromeo M.D., Savage T.K., Kollipara R.K., He M., Augustyn A., Osborne J.K., Girard L., Minna J.D., Gazdar A.F., Cobb M.H., Johnson J.E. (2016). ASCL1 and NEUROD1 Reveal Heterogeneity in Pulmonary Neuroendocrine Tumors and Regulate Distinct Genetic Programs. Cell Rep..

[58] Choudhury N.J., Lai W.V., Makhnin A., Heller G., Eng J., Li B., Preeshagul I., Santini F.C., Offin M., Ng K. (2024). A Phase I/II Study of Valemetostat (DS-3201b), an EZH1/2 Inhibitor, in Combination with Irinotecan in Patients with Recurrent Small-Cell Lung Cancer. Clin. Cancer Res..

[59] Rudin C.M., Reck M., Johnson M.L., Blackhall F., Hann C.L., Yang J.C.H., Bailis J.M., Bebb G., Goldrick A., Umejiego J., Paz-Ares L. (2023). Emerging therapies targeting the delta-like ligand 3 (DLL3) in small cell lung cancer. J. Hematol. Oncol..

[60] Paul S., Konig M.F., Pardoll D.M., Bettegowda C., Papadopoulos N., Wright K.M., Gabelli S.B., Ho M., van Elsas A., Zhou S. (2024). Cancer therapy with antibodies. Nat. Rev. Cancer.

[61] Shen L., Sun X., Chen Z., Guo Y., Shen Z., Song Y., Xin W., Ding H., Ma X., Xu W. (2024). ADCdb: the database of antibody-drug conjugates. Nucleic Acids Res..

[62] DeLucia D.C., Cardillo T.M., Ang L., Labrecque M.P., Zhang A., Hopkins J.E., De Sarkar N., Coleman I., da Costa R.M.G., Corey E. (2021). Regulation of CEACAM5 and Therapeutic Efficacy of an Anti-CEACAM5-SN38 Antibody-drug Conjugate in Neuroendocrine Prostate Cancer. Clin. Cancer Res..

[63] Du Y., Wen Y., Huang J. (2024). Analysis of variation of serum CEA, SCC, CYFRA21-1 in patients with lung cancer and their diagnostic value with EBUS-TBNA. J. Med. Biochem..

[64] Yu M., Wang P. (2024). A comparative study of ProGRP and CEA as serological markers in small cell lung cancer treatment. Discov. Oncol..

[65] Kim Y.S., Nabet B.Y., Cortez B.N., Sun N.Y., Sebastian R., Redon C.E., Ray A., Liu L., Ishola A.A., Loew S. (2025). NOTCH1 reverses immune suppression in small cell lung cancer through reactivation of STING. J. Clin. Investig..

[66] Roper N., Velez M.J., Chiappori A., Kim Y.S., Wei J.S., Sindiri S., Takahashi N., Mulford D., Kumar S., Ylaya K. (2021). Notch signaling and efficacy of PD-1/PD-L1 blockade in relapsed small cell lung cancer. Nat. Commun..

[67] Mayakonda A., Lin D.C., Assenov Y., Plass C., Koeffler H.P. (2018). Maftools: efficient and comprehensive analysis of somatic variants in cancer. Genome Res..

