# Supplementary material for: SCLC TumorMiner: A genomics platform for small cell lung cancer precision oncology
Source: iScience. 2026 Sep 3;29(9):117376. doi: 10.1016/j.isci.2026.117376 (PMC13571741; doi:10.1016/j.isci.2026.117376)
Supplement: Supplementary file 1 [file mmc1.pdf]

**iScience, Volume *29***

## Supplemental information

# SCLC TumorMiner: A genomics platform for small cell lung cancer precision oncology

**Fathi Elloumi, Anjali Dhall, Daiki Taniyama, Augustin Luna, Yasuhiro Arakawa, Sudhir Varma, Yanghsin Wang, Anisha Tehim, Mark Raffeld, Kenneth Aldape, Christophe Redon, Roshan Shrestha, William Reinhold, Mirit Aladjem, Jaydira Del Rivero, Nitin Roper, Anish Thomas, and Yves Pommier**

# Supplemental Figures

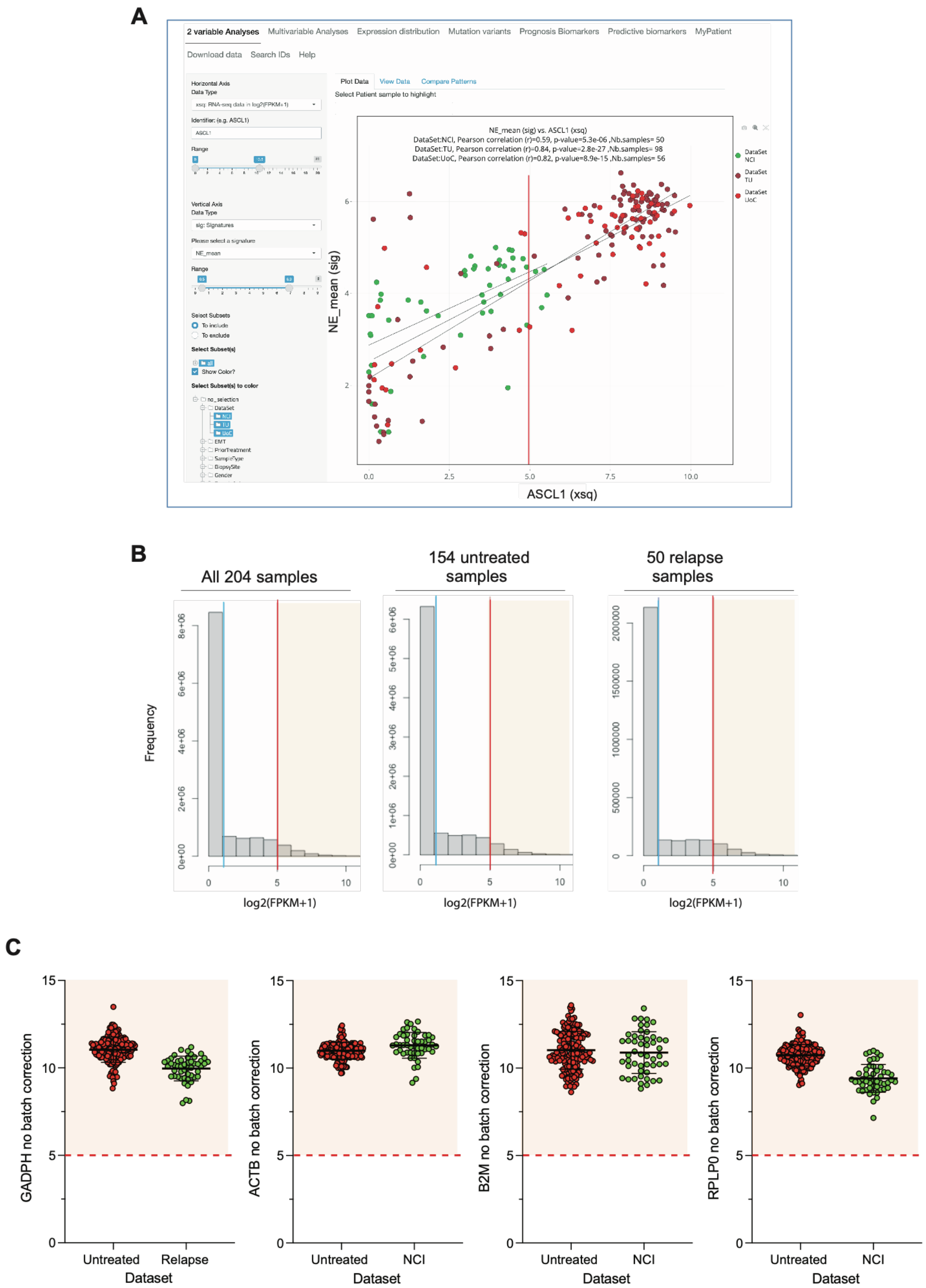


**Figure S1 Gene expression in untreated and relapse cohorts**. **A**. Example of a "2 Variable Analysis" snapshot of the websites displaying the correlation between *ASCL1* expression and the NE signature. **B**. Frequency distribution of gene expression across the datasets. The red line indicates the >90% cutoff (log2(FPKM+1)>5) for high gene expression. **C**. Comparative expression of 4 canonical housekeeping genes in the untreated and relapse samples. Colored areas define samples with significant expression ((log2(FPKM+1) > 5.0 in pink). Data were downloaded from the SCLC TumorMiner website using the download tabs.

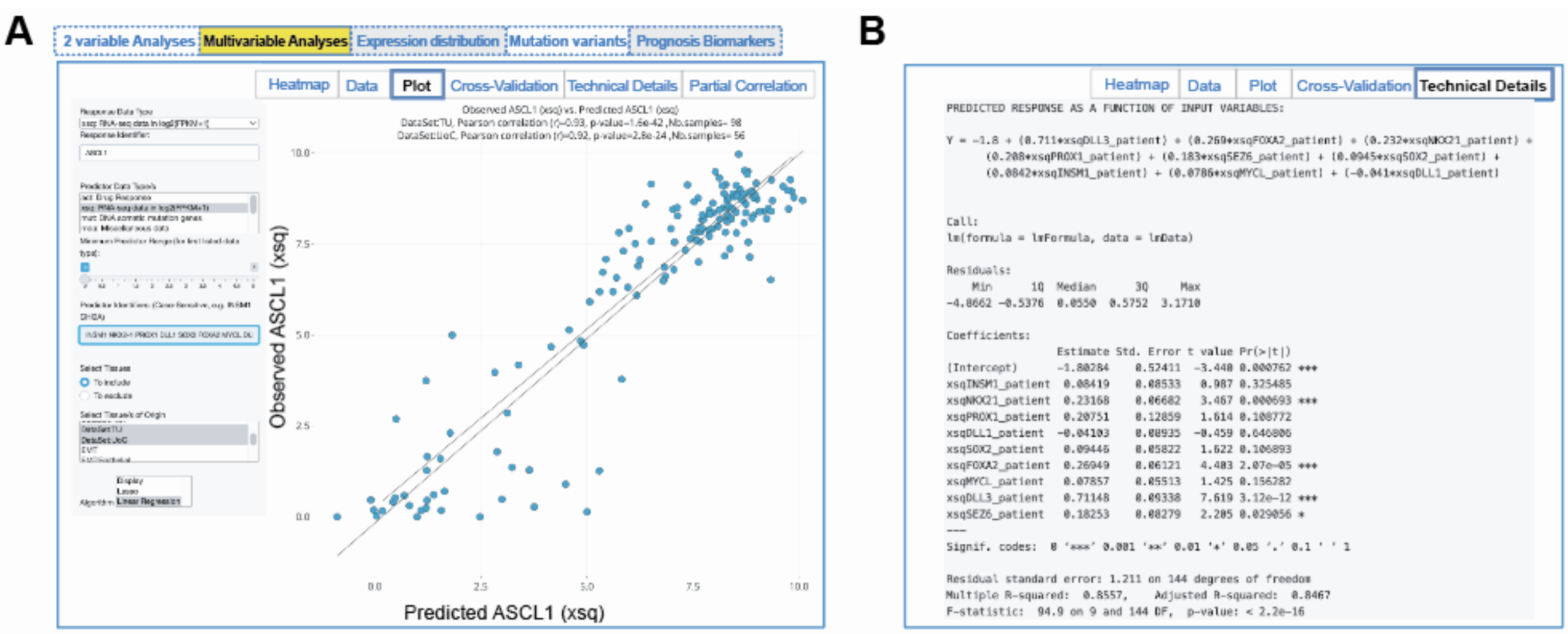


**C**

Predictive value for the listed Response Identifiers

| Response identifier | **ASCL1** (panel A) | **ASCL1** (panel B) | **NEUROD1** (panel C) | **POU2F3** (panel D) | **YAP1** (panel E) | **REST** (panel F) |
|---|---|---|---|---|---|---|
| Prediction **NCI** | r=0.77; p=8.8e-11 | r=0.91; p=1.6e-20 | r=0.86; p=1.6e-15 | r=0.90; p=1.2e-18 | r=0.91; p=9.3e-20 | r=0.90; p=03.6e-19 |
| Prediction **TU** | r=0.89; p=8.2e-34 | r=0.93; p=1.1e-43 | r=0.85; p=4.6e-28 | r=0.97; p=4.2e-64 | r=0.86; p=1.3e-29 | r=0.93; p=2.5e-42 |
| Prediction **UoC** | r=0.90; p=3.3e-21 | r=0.94; p=1.9e-27 | r=0.91; p=2.5e-22 | r=0.96; p=2.0e-29 | r=0.91; p=1.3e-22 | r=0.94; p=1.7e-27 |

(r) = Pearson correlation

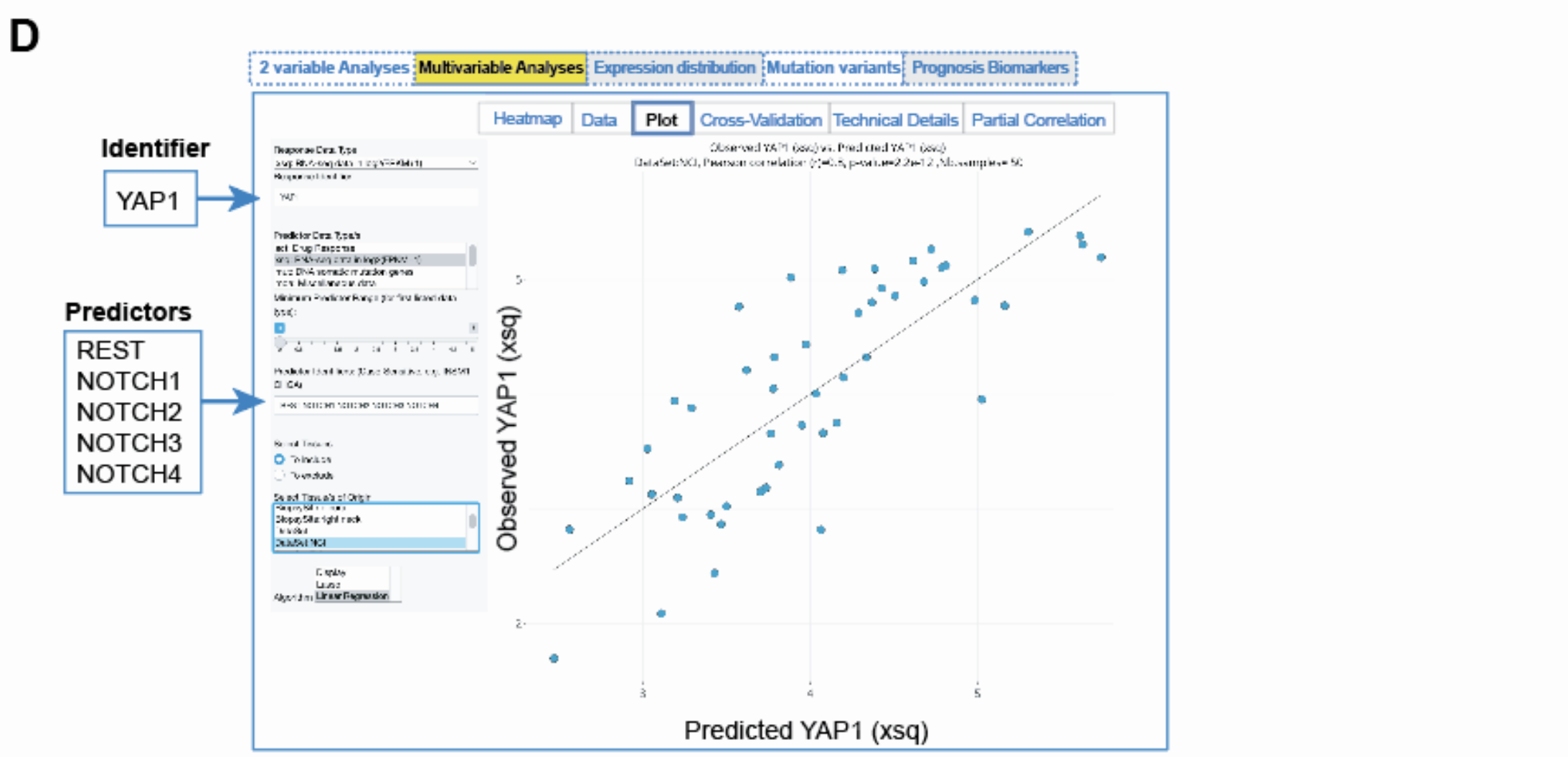


**Figure S2 NAPY predictive models**. **A**. Snapshot of the plot corresponding to the predictive value of the 8 variables ("Predictor Identifiers") listed in Figure 2A under "Multivariable Analyses". **B**. Snapshot of the "Technical Details" corresponding to the plot shown in panel A and the Heatmap shown in Figure 2A. **C**. Linear regression analyses for the Heatmaps shown in panels A-F of Figure 2. Pearson correlation coefficients and p-values were obtained from the "Plot" tab (as shown in panel A for Figure 2A). **D**. Expression of the Notch pathway canonical genes *REST*, *NOTCH1*, *NOTCH2*, *NOTCH3* & *NOTCH4* predict the expression of *YAP1*.

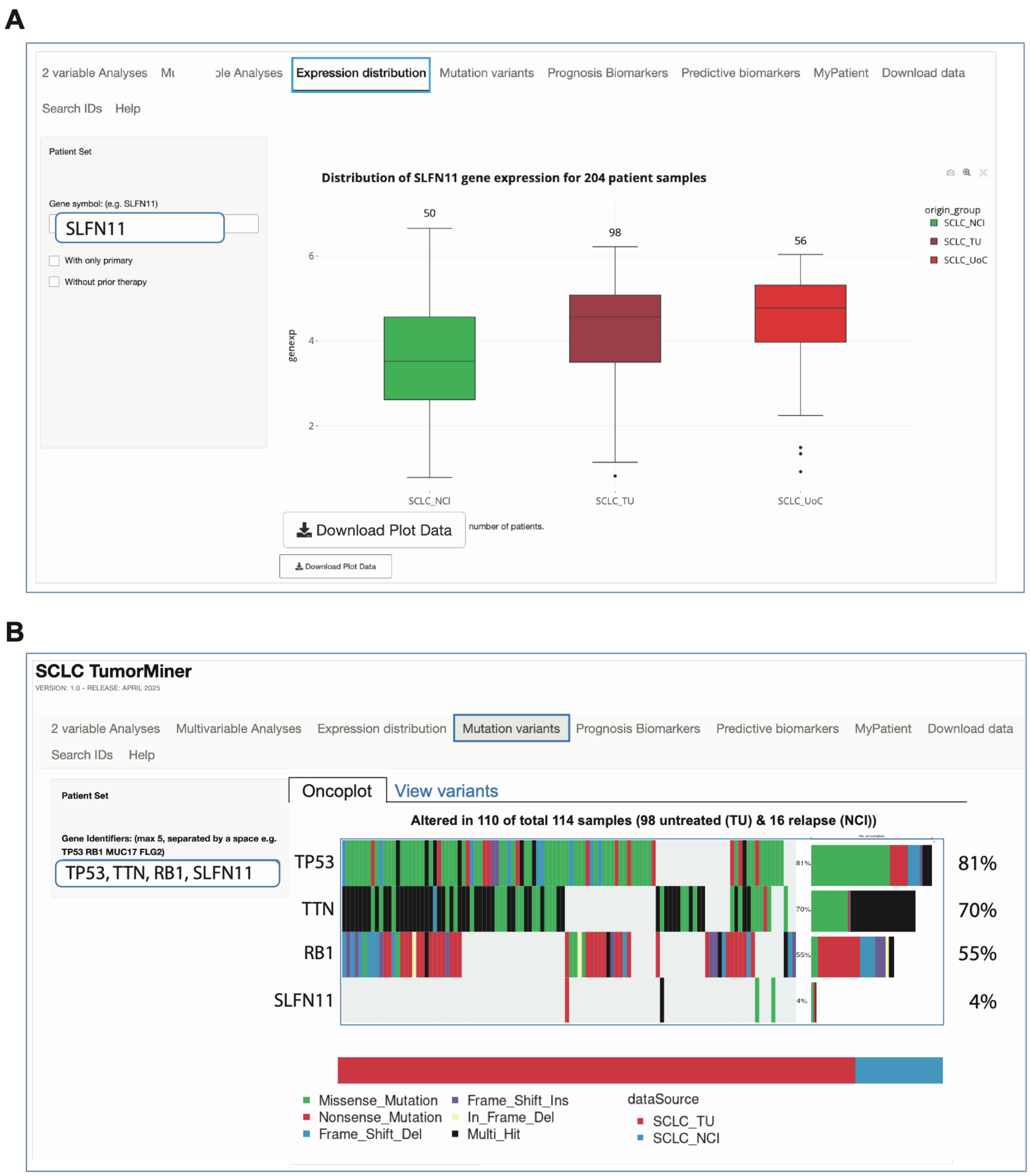


**Figure S3 Gene expression and variant mutation use cases**. **A**. Snapshot of the "Expression distribution" tab from the SCLC TumorMiner website in each of the 3 datasets of SCLC TumorMiner. Gene expression (genexp) is expressed as log2(FPKM+1). Statistical comparisons are performed by Student t-test. **** indicates p < 0.001; ** p < 0.01; * p < 0.05. The "Download Plot Data" tab at the bottom left enables users to export the data. **B**. Snapshot of the "Mutation Variants" tab in the SCLC TumorMiner website. Number to the right indicate the percentage of samples with deleterious mutations.

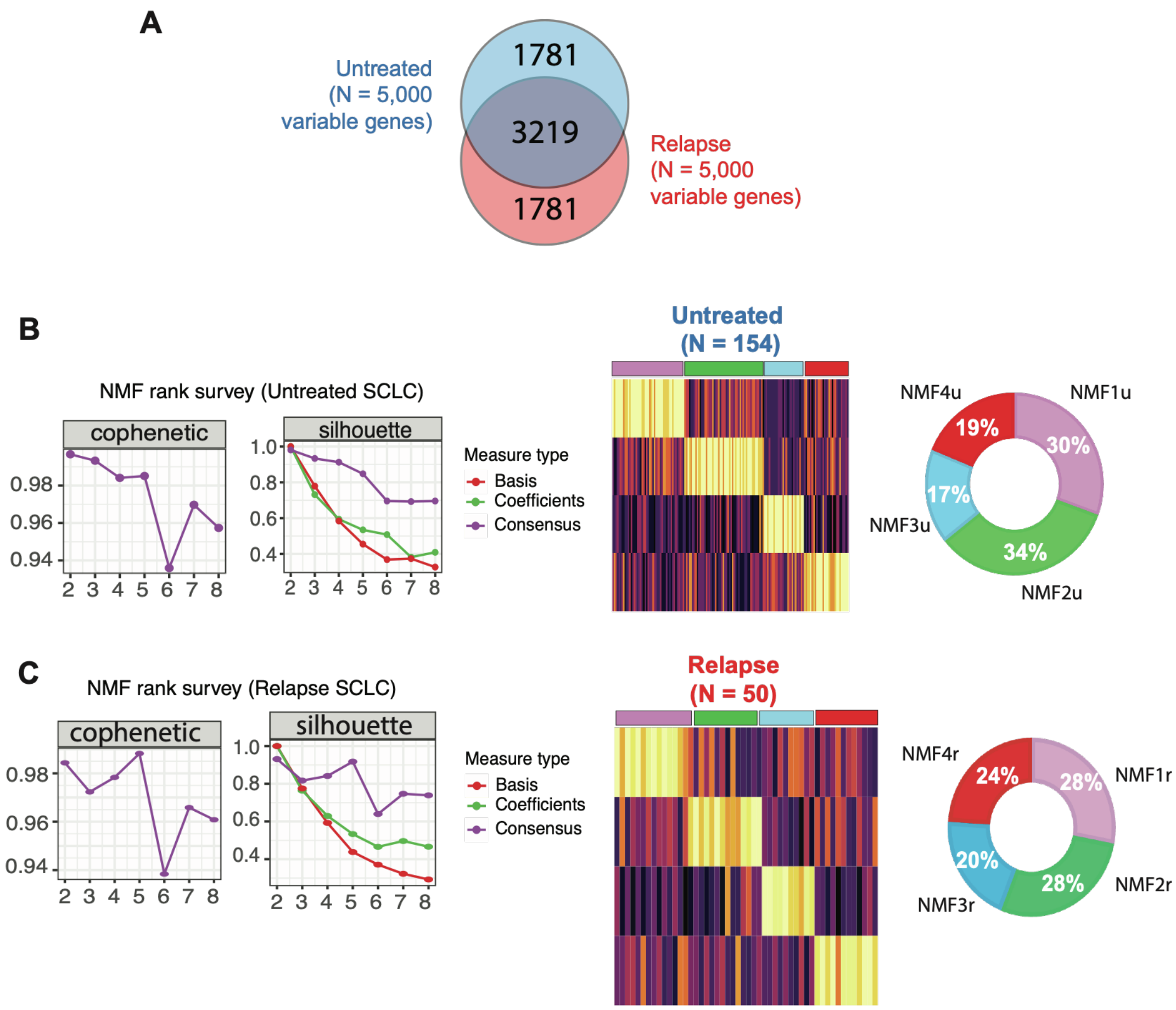


**Figure S4 NMF metrics**. **A**. Venn-diagram showing the overlap of Untreated (top 5000) and Relapse (top 5000) most variable genes, selected using Median Absolute deviation (MAD) approach. **B** and **C** NMF rank summary for untreated and relapse SCLC samples, respectively. The cophenetic and silhouette scores are plotted for range of clusters (k=2:8), depicting k=4 as optimal number of clusters for both cohorts. Consensus matrix heatmaps at k=4 show well defined and stable clusters. Right: pie charts showing the distribution of samples in each NMF cluster.

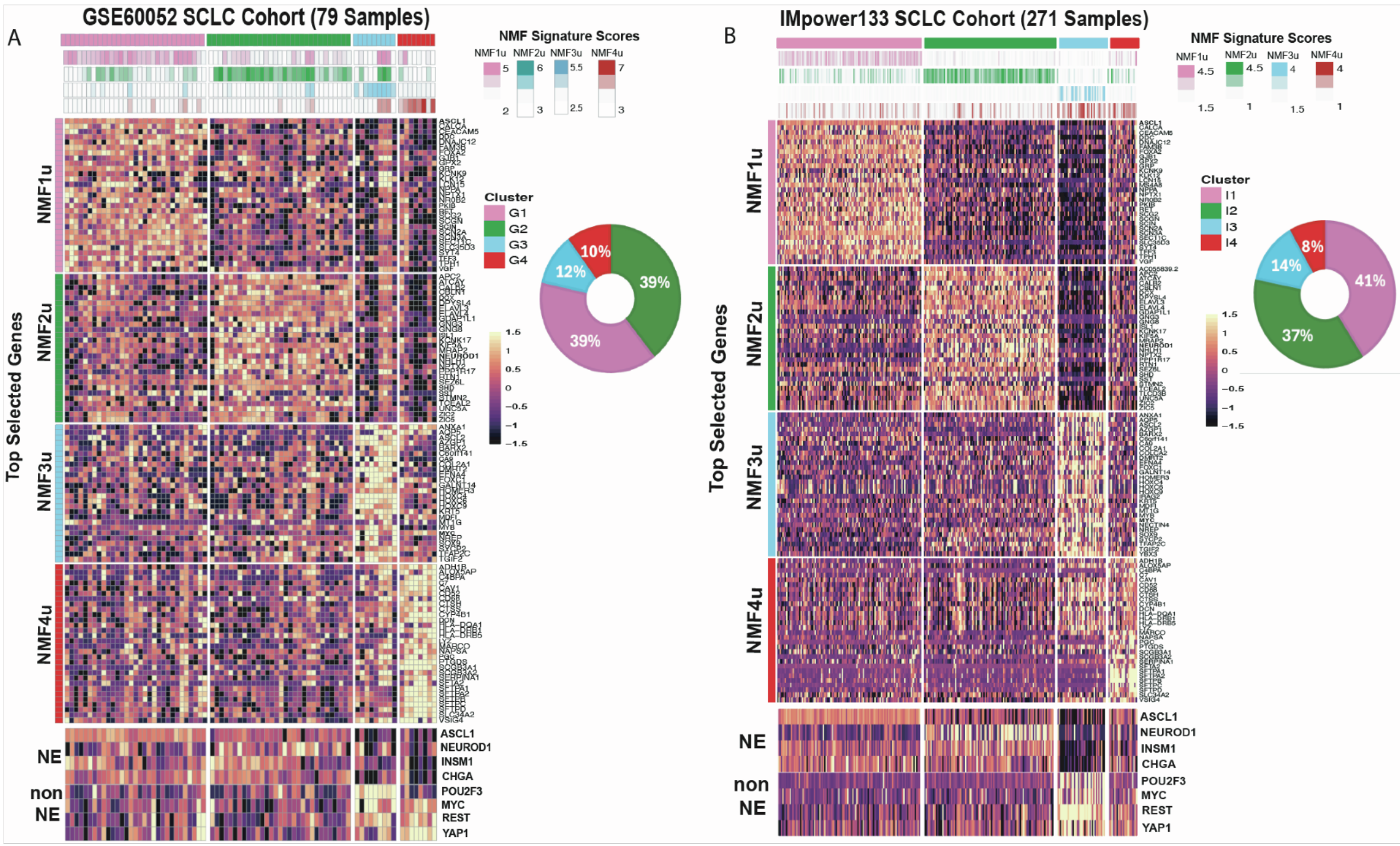


**Figure S5 External validation of the NMFu derived gene signature on two independent SCLC cohorts (GSE60052 and Impower133).** A. Heatmap for Cohort GSE60052. Each column represents a SCLC sample, and rows show expression of the top-30 most differentially enriched signature genes with colors indicating row-scaled expression levels. The topmost rows show 4 NMF clusters and the NMF signature scores per sample. Pie charts represent the distribution of patient samples across the different clusters. B. Heatmap for the Impowe33 cohort.

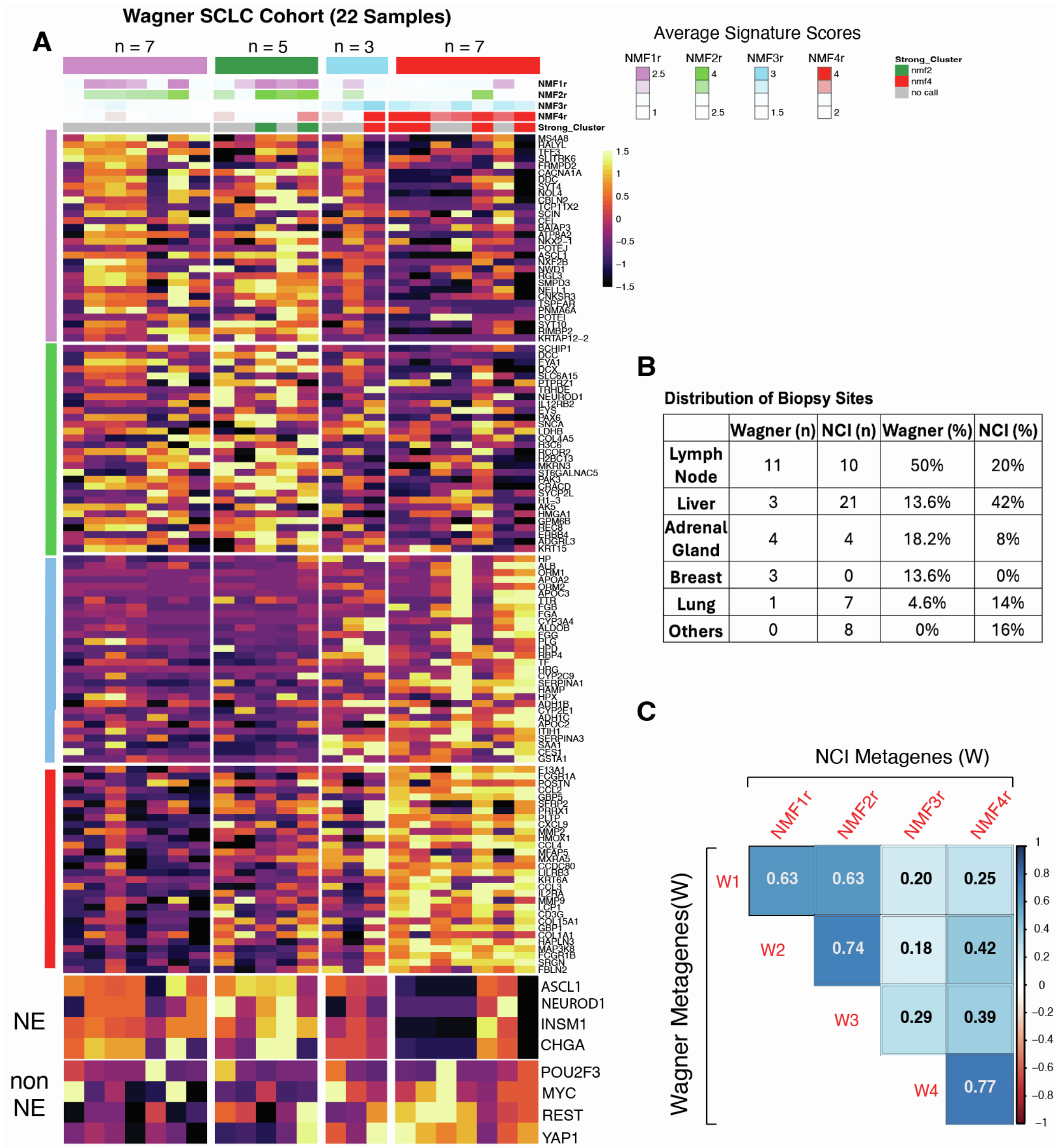


| | Wagner (n) | NCI (n) | Wagner (%) | NCI (%) |
|---|---|---|---|---|
| Lymph Node | 11 | 10 | 50% | 20% |
| Liver | 3 | 21 | 13.6% | 42% |
| Adrenal Gland | 4 | 4 | 18.2% | 8% |
| Breast | 3 | 0 | 13.6% | 0% |
| Lung | 1 | 7 | 4.6% | 14% |
| Others | 0 | 8 | 0% | 16% |

**Figure S6 External validation of the NMFr-derived gene signature in an independent relapse SCLC cohort (Wagner et al. cohort)** [27]. **A**. Heatmap showing the expression of the top-30 most differentially enriched genes from the NCI NMFr-derived signature across the relapse samples of the Wagner et al. cohort (n = 22). Columns represent individual tumor sample, grouped by NMF cluster assignment. Rows represent the differentially enriched gene. Colors indicate row-scaled expression levels. Top annotations display NMF cluster identity and corresponding signature scores (NMFr1–NMFr4) for each sample. Bottom annotations indicate neuroendocrine (NE) and non-NE classification based on canonical markers (including *ASCL1*, *NEUROD1*, *POU2F3*, *YAP1*). **B**. Biopsy sites in the Wagner and the NCI cohorts. **C**. Concordance between the Wagner et al. cohort subgroups and NCI metagenes for each of the NMFr. Heatmap shows correlation coefficients between Wagner NMF metagene signatures (rows; W1–W4) and the NCI metagene signatures (columns; NMF1r-NMF4r), demonstrating cross-cohort reproducibility of transcriptional subtypes.

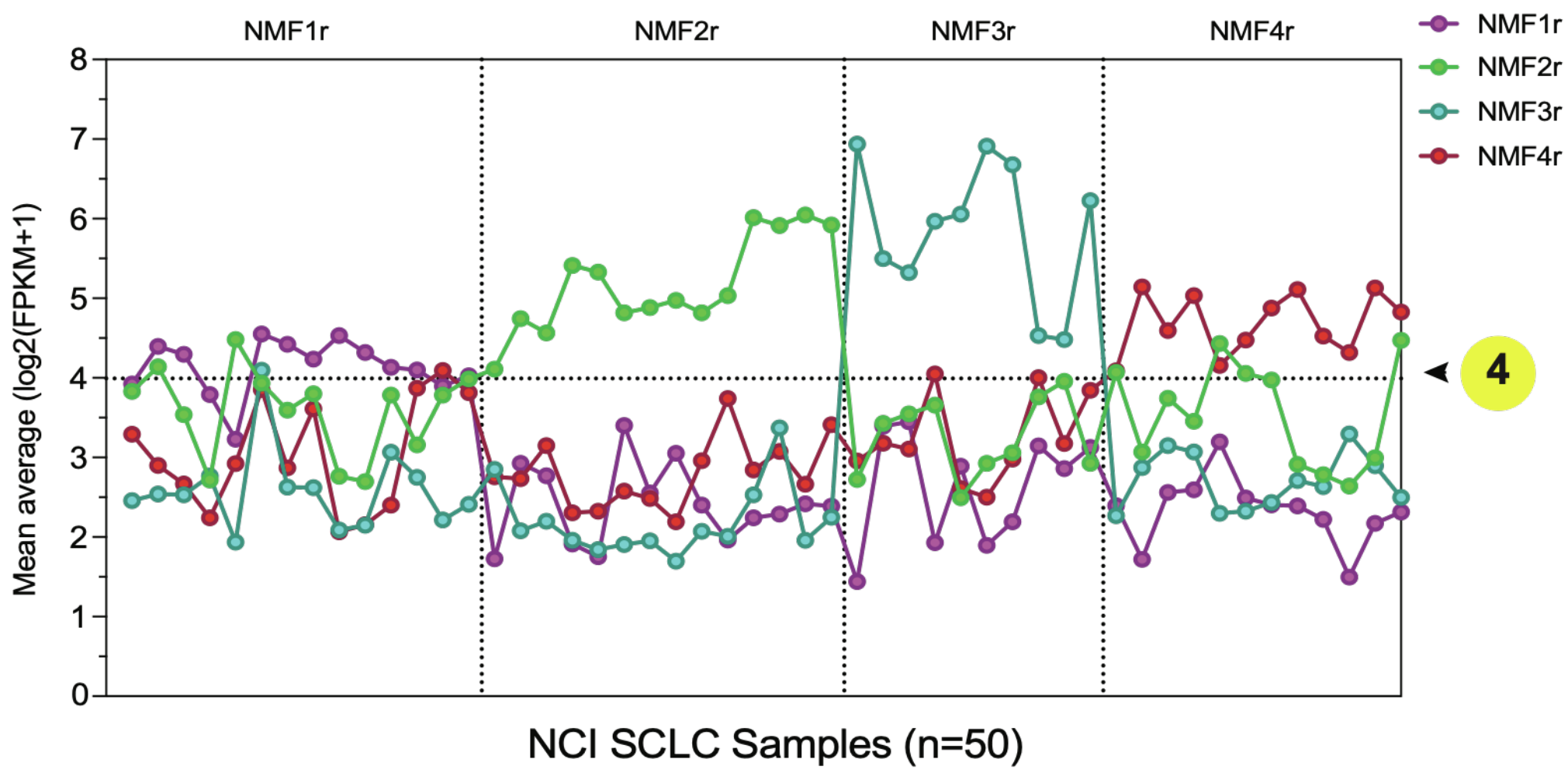


**Figure S7 Mean average NMF expression for each and all 50 samples from patients at relapse**. A mean average threshold value log2(FPKM+1) > 4 is sufficient to assign each sample to one of the 4 NMFs. The gray area defines non-significant values.

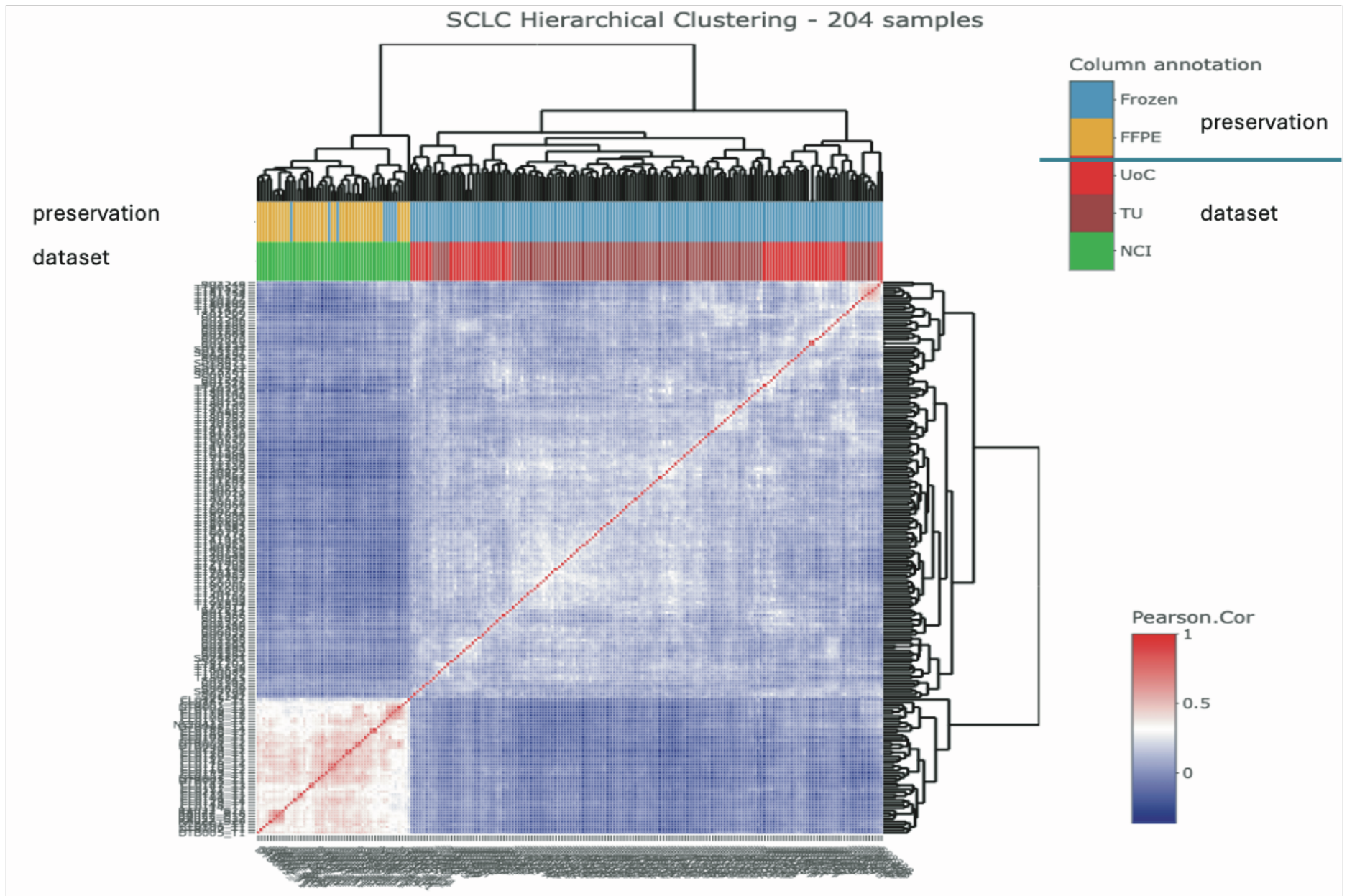


**Figure S8 Hierarchical clustering of SCLC samples**. The clustering shows a clear separation between NCI relapse samples (colored green) and untreated samples (TU, brown; UoC, red), as indicated in the first annotation row (sample type). The second annotation row (preservation method) shows that most NCI relapse samples are FFPE (orange), whereas all untreated samples are frozen (light blue). Because the preservation method is confounded with sample type (relapse vs. untreated), its effect cannot be disentangled.
